# A Parametric Empirical Bayes Approach to Personalized Reference Intervals and Reference Change Values

**DOI:** 10.1093/clinchem/hvaf092

**Published:** 2025-08-22

**Authors:** Eirik Åsen Røys, Kristin Viste, Christopher-John Farrell, Ralf Kellmann, Bashir Alaour, Marit Sverresdotter Sylte, Janniche Torsvik, Heidi Strand, Michael Marber, Torbjørn Omland, Elvar Theodorsson, Graham Ross Dallas Jones, Kristin Moberg Aakre

**Affiliations:** Hormone Laboratory, Department of Medical Biochemistry and Pharmacology, Haukeland University Hospital, Bergen, Norway; Department of Clinical Science, University of Bergen, Bergen, Norway; Hormone Laboratory, Department of Medical Biochemistry and Pharmacology, Haukeland University Hospital, Bergen, Norway; Department of Clinical Science, University of Bergen, Bergen, Norway; Department of Clinical Chemistry, NSW Health Pathology, Liverpool Hospital, NSW, Sydney, Australia; Hormone Laboratory, Department of Medical Biochemistry and Pharmacology, Haukeland University Hospital, Bergen, Norway; King’s BHF Centre of Research Excellence, School of Cardiovascular Medicine and Sciences, King’s College London, London, United Kingdom; Department of Medical Biochemistry and Pharmacology, Haukeland University Hospital, Bergen, Norway; Gade Laboratory for Pathology, University of Bergen, Bergen, Norway; Multidisciplinary Laboratory Medicine and Medical Biochemistry, Akershus University Hospital, Lørenskog, Norway; Department of Clinical Chemistry, NSW Health Pathology, Liverpool Hospital, NSW, Sydney, Australia; K. G. Jebsen Centre for Cardiac Biomarkers, Institute of Clinical Medicine, University of Oslo, Oslo, Norway; Institute of Clinical Medicine, University of Oslo, Oslo, Norway; Department of Cardiology, Division of Medicine, Akershus University Hospital, Lørenskog, Norway; Department of Biomedical and Clinical Sciences, Division of Clinical Chemistry and Pharmacology, Linköping University, Linköping, Sweden; Department of Chemical Pathology, SydPath, St. Vincent’s Hospital, Sydney, Darlinghurst, NSW, Australia; Faculty of Medicine, University of New South Wales, Kensington, NSW, Australia; Hormone Laboratory, Department of Medical Biochemistry and Pharmacology, Haukeland University Hospital, Bergen, Norway; Department of Clinical Science, University of Bergen, Bergen, Norway; Department of Heart Disease, Haukeland University Hospital, Bergen, Norway

## Abstract

**Background:**

Population-wide reference intervals (RI_pop_) are commonly used in laboratory medicine but may not reflect an individual’s tightly regulated homeostatic interval. Personalized reference intervals (RI_per_) could enhance diagnostic precision by accounting for individual variability. A parametric empirical Bayes (PEB) framework stabilizes individual estimates using population parameters, enabling reliable RI_per_ even from a limited number of individual results.

**Methods:**

We applied the PEB framework to estimate RI_per_ for 9 biomarkers: albumin, creatinine, phosphate, cortisone, cortisol, testosterone, androstenedione, 17-hydroxyprogesterone, and 11-deoxycortisol. The PEB parameters tested were derived from both routine Laboratory Information System (LIS) data and a local biological variation (BV) study. Using serial samples from healthy adults, we assessed the proportion of results flagged with a 95% prediction interval and compared RI_per_ to conventional RI_pop_ and reference change values (RCVs).

**Results:**

LIS parameters were based on data from 1986 to 185 488 patients. PEB-based RI_per_ were consistently narrower than RI_pop_ while maintaining or reducing the proportion of flagged results. For example, albumin flagging decreased from 4.7% (RI_pop_) to 0.3% (RI_per_), phosphate from 5.4% to 3.7%, and cortisone from 7.1% to 3.9%. Conversely, 17-hydroxyprogesterone increased from 0.0% to 5.5% but remained close to the expected 5%. PEB thresholds were narrower than standard RCV estimates by correcting for regression toward the mean without increasing flagged results.

**Conclusions:**

The PEB framework effectively provides personalized cutoffs for laboratory tests even when few individual patient results are available. PEB parameters can be established using LIS or BV data, offering a feasible and cost-effective implementation pathway.

## Introduction

Laboratory medicine has traditionally used population-wide reference intervals (RI_pop_) to interpret diagnostic tests (1, 2). However, this one-size-fits-all approach overlooks an important aspect of physiology: an individual’s homeostatic set point (3). These set points represent tightly regulated, individual-specific biomarker intervals. Recent research shows that these intervals can remain stable over decades and that deviations from one’s baseline are associated with increased mortality (3). Accordingly, shifting to personalized reference intervals (RI_per_) centered on each patient’s set point could enhance diagnostic accuracy (3) and personalize the interpretation of biomarkers.

Coşkun et al. (4, 5) introduced 2 statistical models for establishing RI_per_: one using the population’s average within-subject biological variation (CV_I_) and another using variation derived from an individual’s data. While the latter provides fully personalized thresholds, Coşkun et al. noted that this model typically requires ≥5 samples, limiting feasibility in many clinical settings. In contrast, the CV_I_ model needs fewer samples but assumes a uniform CV_I_ across the population and performs best when the CV_I_ is small relative to the between-subject variation (CV_G_). The reference change value (RCV) is another tool for assessing significant changes between consecutive test results based on CV_I_ (6). While RI_per_ uses multiple data points to establish a patient-specific baseline, RCVs focus on the change between 2 sequential measurements.

McIntosh and Urban’s parametric empirical Bayes (PEB) framework (7) offers a promising alternative for estimating RI_per_ and RCVs based on CV_I_. The PEB is a statistical procedure for estimating individual means drawn from a common population. This framework adjusts the observed individual mean by incorporating information from the population distribution, described by the CV_I_, the CV_G_, and the population mean (μ_pop_). “Borrowing strength” from the population, the model introduces a “shrinkage factor” that shifts the predicted individual set point toward μ_pop_, moderating the influence of individual variability and yielding more stable estimates. The shrinkage factor (B*_n_*), which can be described as improvements in the model estimates with additional data, also accounts for regression toward the μ_pop_; the tendency for extreme values to move closer to the cohort average on repeated testing (8). If unaddressed, this statistical phenomenon can lead to misinterpretation of real changes in serial results. Notably, current RI_per_ methods (4, 5) and RCV calculations (6, 9) do not adjust for regression to the μ_pop_, highlighting a problem that PEB can solve. The parameters used in the PEB can be derived from published biological variation (BV) estimates or real-world data from laboratory information systems (LIS). Routine LIS data offers practical advantages: it inherently includes local preanalytical and analytical variation (CV_A_) and actual clinical sampling patterns (10), providing data that better reflects local practice.

In this study, we have applied the PEB framework to estimate personalized biomarker thresholds across multiple analytes, including those with gaussian and skewed distributions. We have derived PEB-parameters from 2 data sources: a local BV study and routine LIS data. Finally, we evaluated the thresholds and parameters using serial biomarker measurements from healthy individuals.

## Materials and Methods

### The Parametric Empirical Bayes

The PEB framework is related to Bayesian statistics but differs in its treatment of the model parameters. In a Bayesian approach, these parameters are assigned a prior distribution. In contrast, the PEB framework directly estimates the parameters (μ_pop_, within-subject variance σ_I_², and between-subject variance σ_G_²) from the data and then treats them as fixed-point estimates, which can be described as a “frequentist approach.” When predicting an individual’s mean, the PEB assumes that, after appropriate transformation, biomarker values in healthy individuals follow a 2-level hierarchical normal model, where individual observations X*_n_* are independent and identically distributed around the individual’s true mean μ_I_ (7, 11):


(1) $$\begin{array}{rl} & \;\;X_{n}|\;\mu_{I}\overset{i.i.d.}{∼}\;\;N(\mu_{I},\sigma_{I}^{2})\;\mathrm{likelihood},\;\;\\ & \mu_{I}\overset{i.i.d.}{∼}\;\;N(\mu_{\mathrm{pop}},\sigma_{G}^{2})\;\mathrm{prior} \end{array}$$

Under this hierarchical model, the posterior mean $\hat{Y}$ is derived from the conditional expectation of μ_I_ given the individual’s sample mean ${\bar{X}}_{n}$, and the estimated parameters μ_pop,_ σ_G_² and σ_I_². $\hat{Y}$ represents the predicted homeostatic set-point and serves as a compromise between the individual’s observed ${\bar{X}}_{n}$ and the μ_pop_. The PEB provides a closed-form approximation of $\hat{Y}$ in Formulae 2 and 3:


(2) $$\;\hat{Y}=\mu_{\mathrm{pop}}+({\bar{X}}_{n}\;-\;\mu_{\mathrm{pop}})\cdot B_{n}$$

(3) $$\;B_{n}=\;\frac{\sigma_{G}^{2}}{\sigma_{G}^{2}+\;\frac{\sigma_{I}^{2}}{n}}$$

$\hat{Y}$ is calculated as a weighted average of ${\bar{X}}_{n}$ and μ_pop_, with the weighting determined by the degree of individual variability through the B*_n_*. B*_n_* is between 0 and 1 depending on the ratio of σ_G_² to σ_I_² and *n* number of previous individual results. For example, when σ_I_² is high and only a few individual results are available, the likelihood of regression toward the μ_pop_ increases (8, 9). Under these conditions, we see from formula 3 that B*_n_* approaches 0, which reduces weighting on the factor (${\bar{X}}_{n}\;-\;\mu_{\mathrm{pop}}$ ) shifting $\hat{Y}$ closer to μ_pop_, thereby accounting for this effect. Mcintosh and Urban (7) noted that this adjustment reduces the variance of $\hat{Y}$ compared to ${\bar{X}}_{n}$ as the B*_n_* compensates for noise in individual estimates. Consequently, a prediction interval based on $\hat{Y}$ will be narrower than 1 that uses ${\bar{X}}_{n}$ to estimate the homeostatic set point (7), as in the current CV_I_-based RI_per_ method (4). The PEB prediction interval for significant change in the subsequent individual measurement (X*_n_*_+1_) is derived from variance of the difference X*_n_*_+1_ – $\hat{Y}$ (7):


(4) $$|X_{n+1}-\hat{Y}|>\;Z\;\cdot \sqrt{1-B_{1}\cdot B_{n}\;}\cdot \sigma_{\mathrm{pop}}$$

Here, Z is the Z-score specifying the confidence level, σ_pop_ is the estimated population's SD equal to √(σ_G_² + σ_I_²), and B_1_ equals B*_n_* at *n* = 1. The intraclass correlation (B_1_) can also be used to re-express B*_n_*:


(5) $$B_{n}=\;\frac{B_{1}\cdot n\;\;}{B_{1}\cdot n+(1-B_{1})}\;$$

Formula 5 is an extension of formula 3 that includes the case *n* = 0 (see proof 1 in Supplemental Formulae). Specifically, at *n* = 0, B*_n_* = 0 and $\hat{Y}$ = μ_pop_, making the threshold |X_1_ - μ_pop_| > Z · σ_pop_. This threshold equals the RI_pop_, meaning all subjects share the same threshold for the first individual measurement. Conversely, as *n* → ∞, B*_n_* ≈ 1, $\hat{Y}\approx {\bar{X}}_{n}$, and the threshold converges to $\left|X_{n+1}-{\bar{X}}_{n}\right|$ > Z · σ_I_ (see proof 2 in Supplemental Formulae). This defines the RI_per_ and transitions smoothly from RI_pop_. In other words, as more individual results become available, the thresholds become centered at the individual mean. For the case of significant change in pairs of results (n = 1), the PEB conceptually corresponds to the RCV but provides narrower thresholds (see proof 3 in Supplemental Formulae). Finally, since $\sqrt{1-B_{1}\cdot B_{n}\;}\;<\;1$ for any *n* ≥ 1, formula 4 shows that personalized PEB thresholds are always narrower than RI_pop_. In summary, the PEB integrates the RI_pop_, RCV, and RI_per_ concepts within a unified framework determined by the number of previous results considered. By incorporating the B*_n_*, PEB consistently provides tighter cutoffs than traditional RCV or RI_per_ calculations. To implement the PEB in practice, one must first estimate the 3 parameters: B_1_, μ_pop_, and σ_pop_, which we aim to do from both LIS and BV data.

### Data Collection and Analysis

Data were collected in accordance with the Declaration of Helsinki Ethical Principles and Good Clinical Practice.

#### LIS Dataset

We used a previously established dataset of adult patients (18–110 years) from the LIS (July 2015–July 2023) to estimate PEB parameters. The extraction of patient results from the LIS without patient consent was approved by the Regional Committee for Medical and Health Research Ethics in Bergen (ID number 252 125). The selected biomarkers (chosen to represent different levels and distributions of BV) included 11-deoxycortisol (11-DOC); 17-hydroxyprogesterone (17-OHP); testosterone; androstenedione; and cortisone analyzed on a locally developed steroid panel (12) using LC-MS/MS instruments from Agilent, AbSciex, or Waters. Long-term CV_A_ is shown in Table 1. The steroid panel data included both clinically requisitioned results and unrequested results that were automatically generated as part of the panel analysis. We excluded the clinically requisitioned results and dynamic function tests. The remaining unrequested results were primarily from men (>90%), so we focused on the male subgroup (excluding results from women) for these markers. Additionally, we included albumin, creatinine, and phosphate results from a Cobas 8000 analyzer (Roche Diagnostics). All biomarker values below the reporting limit were excluded (Supplemental Table 1). To account for diurnal variation, we only retained samples collected between 8:00 Am and 10:00 Am for 11-DOC, 17-OHP, androstenedione, cortisol, cortisone, and creatinine.

**Table 1.** Characteristics of the PEB parameters derived from LIS data and BV estimates.^a^

		LIS parameters (Box-Cox scale)									
		refineR reference interval				Robust regression		BV parameters (original scale)			
Measurand	Subgroup	Samples	μ_pop_	σ_pop_	λ	Result pairs	B_1_ (95% CI)	CV_G_ (95% CI)	CV_I_ (95% CI)	CV_A_	μ_pop_
Albumin (g/dL)	All	185 488	3.181	0.241	0.890	930 184	0.66 (0.66, 0.66)	6.0 (4.7, 8.1)	2.3 [2.1, 2.6]	1.8	4.522
Creatinine (mg/dL)^b^	All	115 632	−0.182	0.211	0.081	457 784	0.92 (0.92, 0.92)	16.0 (12.4, 22.2)	4.4 [4.0, 4.8]	3.0	0.833
Phosphate (mg/dL)^c^	All	10 931	2.098	0.467	0.854	27 836	0.58 (0.57, 0.59)	13.6 (10.7, 18,6)	9.5 [8.7 10.4]	1.7	3.327
Cortisone (µg/dL)^d^	Male	4989	0.626	0.263	0.427	6760	0.50 (0.48, 0.53)	15.2 (10.7, 26.7)	12.6 [11.0, 15.0]	7.0	1.742
Cortisol (µg/dL)^d^	Male	3336	6.297	1.586	0.655	4910	0.43 (0.40, 0.45)	11.7 (6.4, 28.3)	15.1 [12.5, 18,6]	4.4	12.11
Testosterone (ng/dL)^d^	Male	1986	8.989	0.957	0.136	3252	0.76 (0.73, 0.78)	29.0 (21.7, 51.9)	15.3 [13.3, 17.6]	4.1	356.0
Androstenedione (ng/dL)^d^	Male	4765	7.791	1.498	0.284	7244	0.62 (0.60, 0.64)	26.1 (18.7, 47.1)	19.2 [17.1, 22.6]	5.3	60.99
17-OHP (ng/dL)^d^	Male	4712	13.78	4.605	0.507	7171	0.71 (0.69, 0.73)	24.9 (17.0, 45.9)	23.0 [20.1, 27.0]	4.8	60.13
11-DOC (ng/dL)^d^	Male	4905	4.201	1.144	0.108	7514	0.46 (0.43, 0.48)	33.5 (20.4, 62.9)	50.0 [44.9, 61.1]	5.3	31.91

^a^The LIS parameters are established on the Box-Cox scale, which allows for negative values after transformation (e.g., for creatinine), where the λ specifies the transformation applied to normalize the biomarker distributions. The PEB parameters include the population mean (μ_pop_), population SD (σ_pop_), and intraclass correlation (B_1_), as well as CV_G_ and CV_I_, both reported with 95% CIs. The long-term CV_A_ is derived from internal quality control data. The μ_pop_ of the BV parameters are the refineR μ_pop_ backtransformed from the Box-Cox scale to the original scale.

^b^Original creatinine concentrations were measured in µmol/L and converted to conventional units using a factor of 1 µmol/L equaling 0.0113 mg/dL.

^c^Phosphate concentrations were originally measured in mmol/L and converted using a factor of 1 mmol/L equaling 3.1 mg/dL.

^d^Hormone concentrations were reported in nmol/L, with the following conversion factors: cortisone, 1 nmol/L = 0.0360 µg/dL; cortisol, 1 nmol/L = 0.0362 µg/dL; testosterone, 1 nmol/L = 28.8 ng/dL; androstenedione, 1 nmol/L = 28.6 ng/dL; 17-OHP, 1 nmol/L = 33 ng/dL; and 11-DOC, 1 nmol/L = 34.7 ng/dL.

#### Estimating PEB Parameters from the LIS Data

As Urban and McIntosh suggested (7), $\hat{Y}$ can be interpreted as regressing repeated measurements where previous results predict the next (i.e., regressing ${\bar{X}}_{n}$ and X*_n_*_+1_). In this context, the regression slope between result pairs (*n* = 1) corresponds to B_1_. The μ_pop_ and σ_pop_ are equal to the mean and SD of the RI_pop_. The PEB parameters can, in other words, be estimated from the regression of patient result pairs and the local reference interval. This approach assumes (*a*) the data approximate normality after transformation and (*b*) robustness to the influence of pathological values on the parameter estimates. We combined linear regression with the “refineR” algorithm (13) to ensure that our LIS data met these assumptions. RefineR, widely used to derive RI_pop_ from LIS data (14), isolates a “central peak” representing nonpathological results. These results are presumed to constitute most of the dataset (ideally >70%) and to follow—or be transformable into—a Box-Cox normal distribution. The Box-Cox transformation is a robust approach to make skewed data approximate a normal distribution (15). Accordingly, the characterized central peak is taken to represent the nonpathological portion of the dataset.

**Fig. 1.**
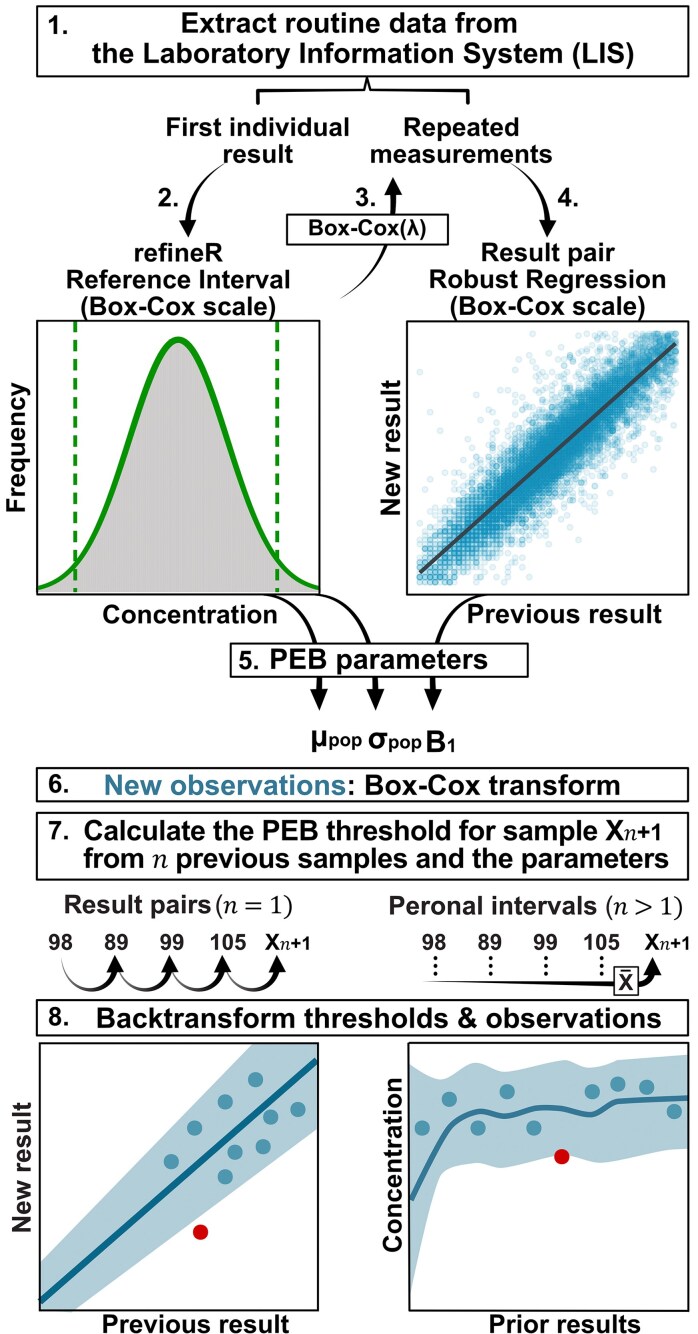
Workflow for establishing and applying LIS PEB parameters: (1) extract LIS data; (2) apply refineR to the first individual result; (3) filter result outside the central 99% distribution defined by refineR and transform the remaining data using the refineR Box-Cox (λ); (4) define result pairs from repeated measurements and perform a robust regression; (5) extract the parameters B_1_ (slope), μ_pop_ and σ_pop_ on the Box-Cox scale; (6) Box-Cox transform new observations using the λ; (7) use the parameters to calculate thresholds for the latest observation (X*_n_*_+1_) based on *n* prior results using the PEB formulae (setting *n* = 1: the previous result is the baseline; setting *n* > 1: the mean of prior results is the baseline; (8) back-transform the established thresholds to the original scale. Color figure available at https://academic.oup.com/clinchem.

Our practical approach to estimating LIS parameters is outlined in the flowchart in Fig. 1. We first applied refineR to the initial results from all patients (minimizing the number of pathological values) to estimate the μ_pop_ and σ_pop_ for each biomarker on the Box-Cox scale. As the refineR model is assumed to represent the nonpathological fraction, we excluded results outside the central 99% distribution. To establish the parameter B_1_, we included the remaining repeated results with >24 h between samples. Data were further Box-Cox transformed using the lambda from refineR (see Supplemental Formulae 1 and 2). We then performed linear regression on patient result pairs to estimate B_1_. Specifically, we used the robust regression “lmrob” in the R package “robustbase” at default settings to minimize the influence of any remaining pathological values. This function iteratively assigns lower weights to observations with large residuals, reducing their impact of outliers without exclusion. We included patients with >2 results in the regression as sequential pairs.

#### Estimating PEB Parameters from BV Studies

To establish PEB parameters from BV data, we used estimates (CV_I_, CV_G_,) from a local state-of-the-art BV study (16). This study was approved by the regional ethics committees at the inclusion sites, the Regional Committees for Medical and Health Research Ethics in Bergen and Oslo (ID: 2018/92), and the South-Central Berkshire Research Ethics Committee (London). All volunteers included in the conventional study gave informed written consent before participating. This study included the same biomarkers, subgroups, and analytical platforms as the LIS data described here. Briefly, this study involved 30 participants (16 males and 14 females) and spanned 10 weeks, with samples collected weekly on the same weekday between 8:00 Am and 10:00 Am (16). A nested-balanced ANOVA with outlier exclusion was used to estimate the CV_I_ and CV_G_, with evaluation of outliers at 4 levels: analytical (Burnett method), participant mean (Reed criterion) (17), individual trend (linear regression, *P* < 0.01), and assessment of CV_I_ homogeneity through the normality of residuals (Shapiro–Wilk test or Bartlett and Cochran methods if data were log-transformed). We excluded outliers until homogeneity was achieved (17) and applied CV-ANOVA or ln-ANOVA (18). BV estimates assume that within-subject variability is proportional to the individual’s mean, yielding a constant CV (19). To align the PEB approach with this assumption, we derived PEB formulae on the log scale (see Supplemental Formulae), where proportional variation is more likely to satisfy the normality assumption. Using these formulae in practice, we combined the CV_I_ and CV_G_ estimates from the BV-study with the long-term CV_A_ estimated from our laboratory’s quality control data (Table 1). For simplicity, we reused the μ_pop_ determined by refineR from the LIS dataset (backtransformed to the original scale) in the formulae.

### Evaluating the Thresholds

**Fig. 2.**
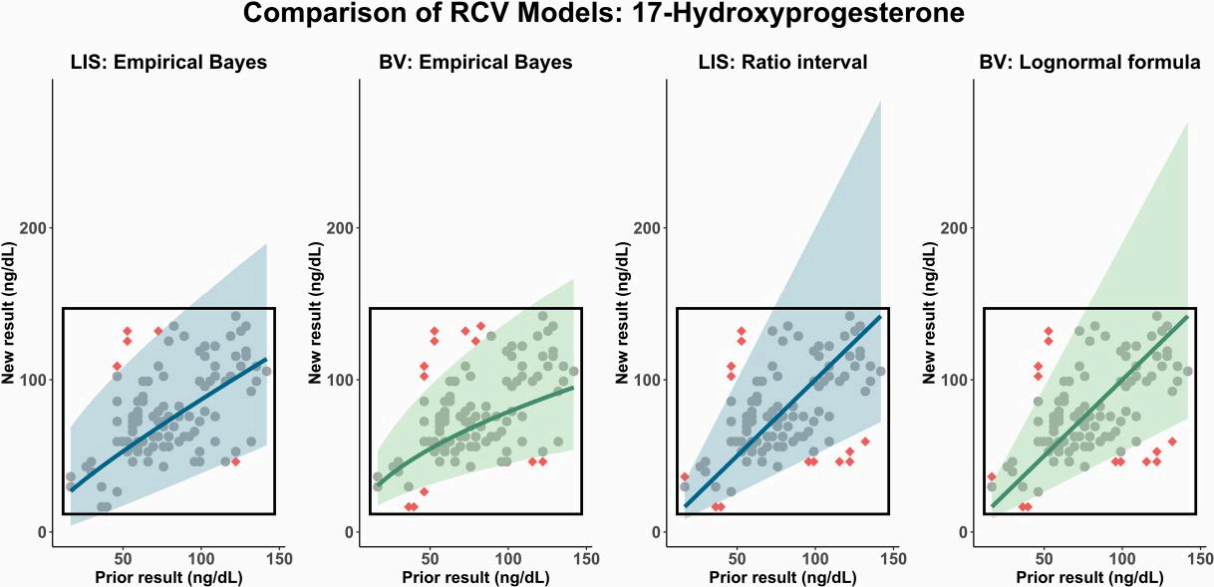
RCVs using a 2-sided 95% prediction interval (shaded area), compared to result pairs (dots) for 17-hydoxyprogesterone from serial measurements of male participants in the biological variation study. The 4 panels compare different RCV models: (1) PEB with parameters based on LIS, (2) PEB with parameters based on BV estimates, (3) ratio interval distributions derived from LIS data, and (4) a BV-based lognormal formula. The diamond shapes indicate flagged result pairs exceeding the RCV thresholds. The central black square is the reference interval estimated by refineR for visual comparison. Color figure available at https://academic.oup.com/clinchem.

To test the validity of the estimated PEB parameters, we compared the proportion of results flagged by RI_pop_, RCVs, and RI_per_ using serial results from the 30 healthy individuals in the BV study. We specified a 2-sided 95% threshold (Z = 1.96), expecting approximately 5% of results to be flagged by adequately calibrated intervals. We applied the PEB algorithm for each participant and biomarker using LIS-based and BV-based parameters. We calculated static RI_pop_ (*n* = 0), result-pair RCVs (*n* = 1), and dynamically updated RI_per_ (incrementing *n* from 0 to 9). The proportion of flagged results was determined by the fraction of an individual’s serial measurements outside each threshold. To investigate if the PEB (*n* = 1) offers an improvement over the standard RCVs, we compared the PEB-derived RCVs to traditionally derived RCVs from our previous study (10) that used either (*a*) the standard ln-normal formula described by Fokkema et al (20) or (*b*) an indirect method based on result ratios from repeat patient measurements in the LIS to establish RCV data. Again, we used the serial measurements of the participants in the BV study with a 2-sided 95% prediction interval to compare the proportion of flagged results for the RCV thresholds (see Fig. 2).

### Software

Data analysis was conducted using R version 4.4.1 with the packages refineR (v.1.6.2), robustbase(v.0.99.4.1), ggplot2(v.3.5.1), and dplyr(v.1.1.4). Scripts with examples are provided at: https://doi.org/10.5281/zenodo.15680355.

## Results

The PEB thresholds are dynamically adjusted based on an individual’s sample mean, and the cutoff values differ for each measurement and patient, making it impractical to summarize personalized thresholds in an aggregate table. Instead, we illustrate the individual thresholds for the individual participants in Figs. 2 to 4 and Supplemental Figs. 1 to 25.

**Fig. 3.**
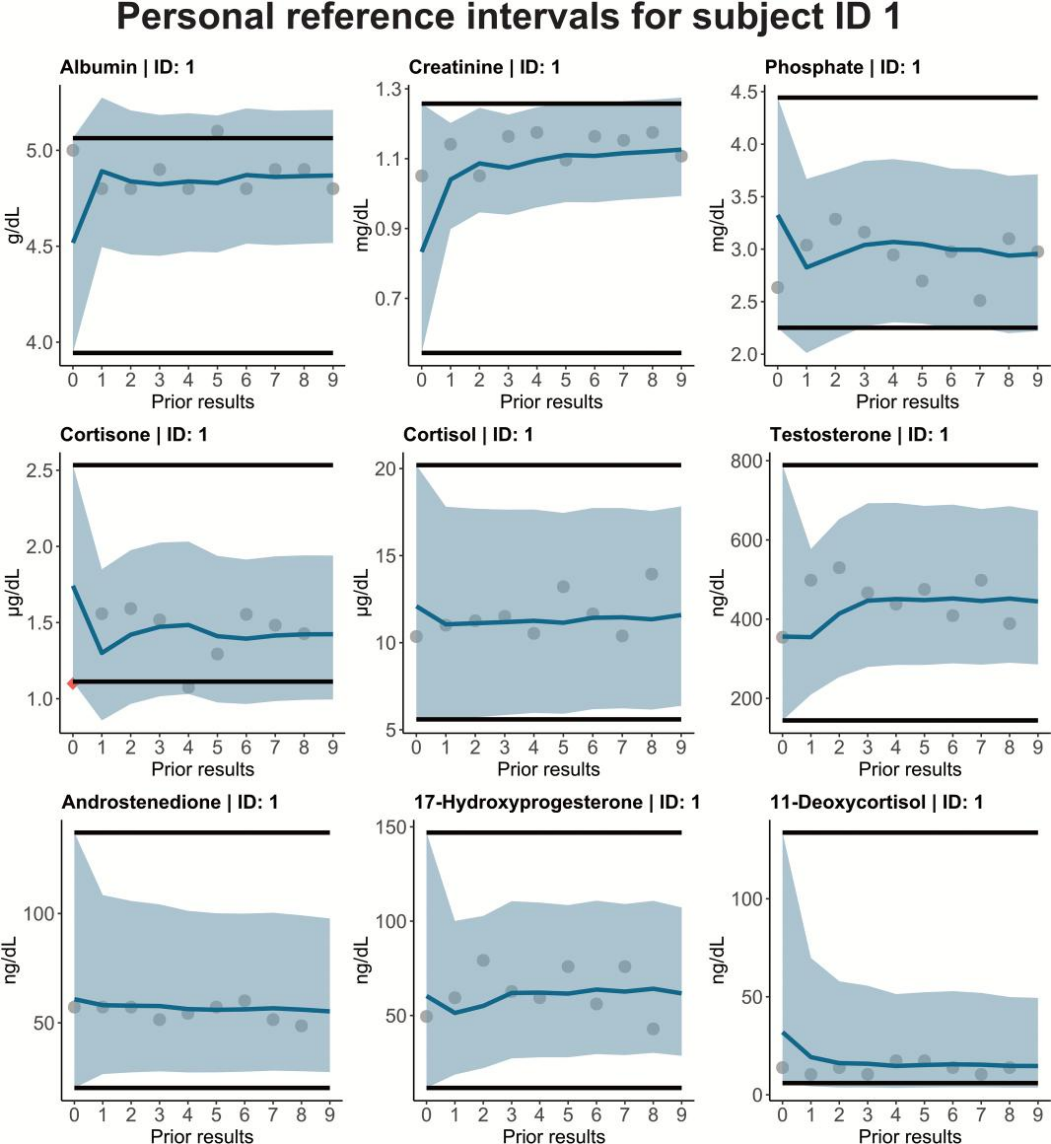
RI_per_ using a 2-sided 95% prediction interval (shaded area), for the first participant in the biological variation study (including all the biomarkers), derived from the PEB method with parameters based on LIS results. Horizontal black lines indicate the 95% RI_pop_ established by the refineR algorithm, while the diamond shape indicates a measurement exceeding the RI_per_. Color figure available at https://academic.oup.com/clinchem.

**Fig. 4.**
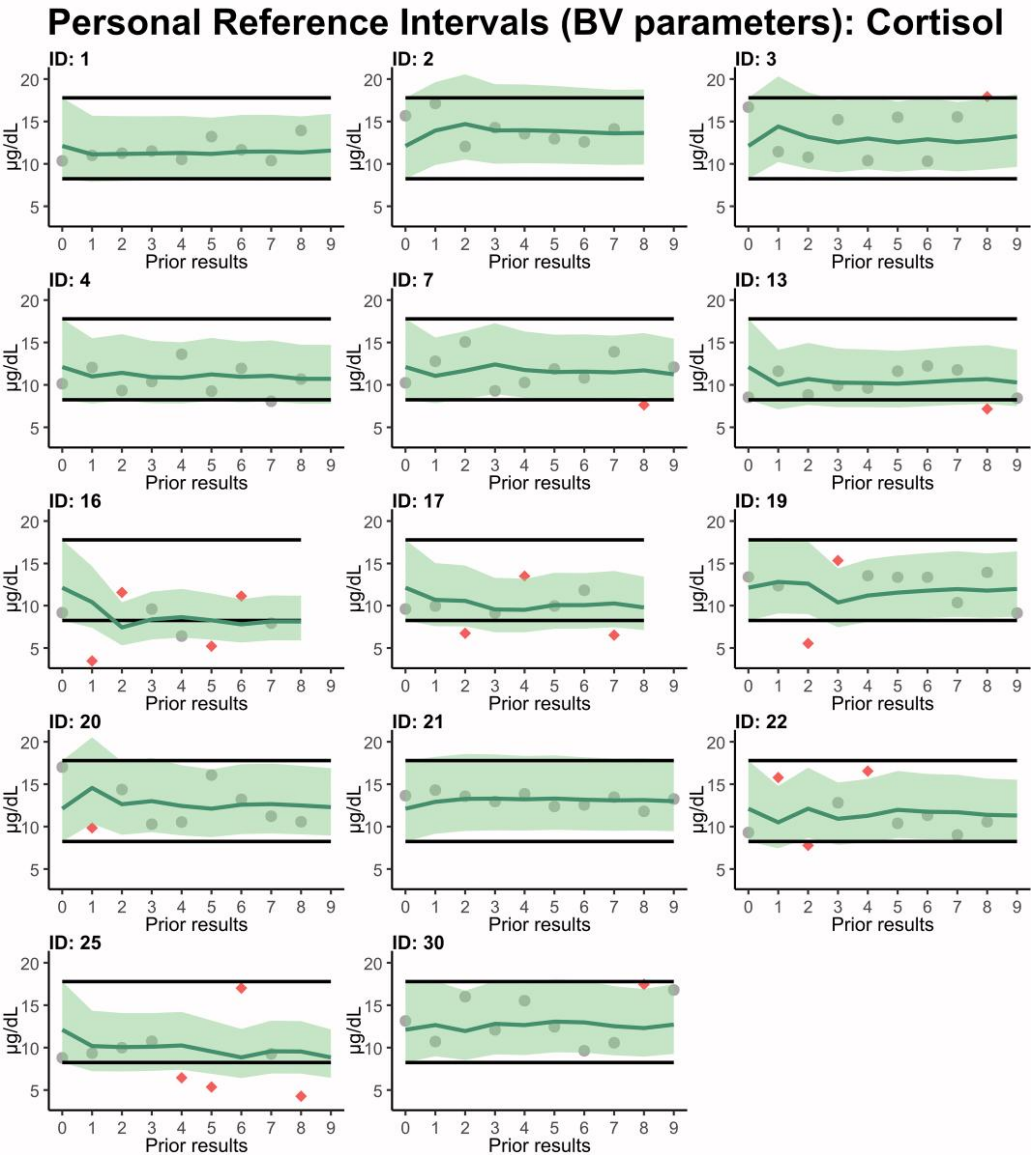
RI_per_ for cortisol, using a 2-sided 95% prediction interval (shaded area), for the males in the BV study derived from the PEB method with parameters based on BV estimates. Horizontal black lines indicate the estimated 95% RI_pop_ from the BV data, while the diamond shape indicates a measurement exceeding the RI_per_. Color figure available at https://academic.oup.com/clinchem.

### Establishing PEB Parameters

Using the refineR algorithm on the LIS dataset, we determined the μ_pop_ and σ_pop_ for each analyte on a Box-Cox–transformed scale (see Table 1 and Supplemental Figs. 26 and 27). We included 1986 (testosterone) to 185 488 (albumin) when applying refineR. To ensure sufficient data for refineR fitting, we included all available testosterone results despite its diurnal variability. We then estimated the B_1_ for each biomarker by regressing result pairs on the Box-Cox scale. The patient result pairs ranged from 3252 (testosterone) to 930 184 (albumin). The resulting B_1_ estimates spanned from 0.419 for cortisol (relatively high CV_I_) to 0.923 for creatinine (relatively high CV_G_); see Supplemental Fig. 28.

Table 1 further summarizes the PEB parameters derived from the BV-study. The CV_G_ ranged from 3.5% (albumin) to 33.0% (11-DOC), while the CV_I_ ranged from 1.7% (albumin) up to 50.0% (11-DOC). In our laboratory, the long-term CV_A_ for the assays was between 1.8% (albumin) and 7.0% (cortisone).

### Flagging of Results

After establishing LIS parameters, we assessed whether the PEB thresholds flagged results at the expected approximately 5% for healthy individuals. As shown in Table 2 and Supplemental Figs. 1 to 9, the LIS parameters RI_per_ maintained equal or lower proportions of flagged results compared with conventional RI_pop_ or RCV. With BV parameters, RI_per_ similarly maintained proportions of flagged results close to the expected 5% (see Table 2 and Supplemental Figs. 17–25), although cortisol (Fig. 4) and 17-OHP notably showed higher proportions of flagged results (16.4% and 11.5%, respectively), suggesting PEB thresholds may not fully capture their true variability.

**Table 2.** Proportion of flagged results evaluated using 2-sided 95% limits: RI_pop_, RI_per_, and RCV based on the PEB approach.^a^

			PEB							
Measurand	Subgroup	Results	RI_pop_ (LIS), %	RI_per_ (LIS), %	RCV (LIS), %	RI_pop_ (BV), %	RI_per_ (BV), %	RCV (BV), %	RCV result ratios (LIS), %	RCV ln-formula (BV)
Albumin	All	298	4.7	0.3	0.4	2.7	5.0	3.0	0.4	3.7%
Creatinine	All	297	0.7	1.0	1.5	5.4	3.0	3.0	3.4	3.4%
Phosphate	All	297	5.4	3.7	3.0	4.7	5.7	6.0	4.5	6.4%
Cortisone	Male	127	7.1	3.9	5.3	7.1	5.5	5.3	5.3	6.2%
Cortisol	Male	128	3.9	3.1	3.5	11.7	16.4	17.5	9.6	17.5%
Testosterone	Male	127	3.1	2.4	3.5	6.3	6.3	9.7	8.8	9.7%
Androstenedione	Male	129	2.3	0.8	2.6	4.7	5.4	7.0	4.3	7.8%
17-OHP	Male	127	0.0	5.5	5.3	17.3	9.4	11.5	12.4	12.4%
11-DOC	Male	120	1.7	1.7	2.8	8.3	7.5	8.4	11.2	6.5%

^a^Estimates are derived from routine patient data (LIS data) and BV estimates from a local study. The proportions of flagged results from the PEB method are compared with those of previously reported RCV thresholds, calculated using LIS data (result ratios) or BV estimates (ln formula).

When comparing RCVs derived from PEB with traditionally derived RCVs from our previous study, we observed fewer flagged results for analytes with larger CV_I_ (see Fig. 2 and Supplemental Figs. 10–17). For instance, the proportion flagged for 17-OHP decreased from 12.4% using traditional ratio-based RCVs to 5.3% with PEB-derived RCVs (LIS parameters).

## Discussion

### Main Findings

Our findings underscore the effectiveness of the PEB in establishing personalized thresholds for clinical laboratory tests across various biomarkers, even when only a limited number of individual results are available. The cutoffs derived from the PEB are consistently narrower than conventional population reference intervals and RCV values. Additionally, we present 2 practical strategies for determining the parameters used in the PEB algorithm: one derived from BV estimates and the other from routine LIS data.

### Personal Reference Intervals

In our study, PEB-based RI_per_ consistently yielded lower proportions of flagged results than standard RI_pop_. These cutoffs better reflect individual physiological ranges and may detect subtle changes. RI_per_ also distributes flagged results more evenly across the cohort. In contrast, RI_pop_ repeatedly flags the same healthy individuals with higher or lower baseline set points. We observed a proportion of flagged results below the intended 5% level for all biomarkers using LIS parameters. We suspect this is due to the greater overall variability in the LIS data compared to the tightly controlled BV study conditions. For example, the RI_per_ for testosterone flagged only 2.4% of results, possibly because we did not filter by diurnal variation for this LIS data. When using BV-derived parameters, we generally observed the intended approximately 5% proportion of flagged results for most biomarkers. Cortisol and 17-OHP, however, showed elevated flagging rates. We suspect that CV_I_ heteroscedasticity, which violates the assumption of the PEB model, is the underlying cause. Seven out of 14 male participants in our BV study were excluded for cortisol before achieving CV_I_ homogeneity (21), introducing substantial selection bias. In contrast, the remaining markers exhibited minimal heteroscedasticity, with at most one individual excluded. Our previous work (16) addressed CV_I_ heterogeneity using a more robust Bayesian model for cortisol and 17-OHP. These findings underscore a fundamental limitation of the PEB approach, which assumes a common CV_I_ across individuals. Consequently, we recommend that laboratories verify RI_per_ locally, consistent with standard practice for RI_pop_ (22).

### RCVs

The traditional RCV calculations do not account for regression toward the mean (9). Consequently, if a patient’s baseline value is far from the μ_pop_, traditional RCVs produce a wide threshold for significant change (8), as shown in Fig. 2. The PEB method addresses this problem by shrinking the expected next value toward the μ_pop_. This adjustment yielded consistently narrower result pair thresholds across all biomarkers in our study. The PEB also flagged fewer results overall compared with the RCVs from our previous work (10). The benefit was most pronounced for analytes with high CV_I_.

### Clinical Application

Personalized thresholds are particularly valuable in clinical scenarios where early disease detection or subtle deviation from a patient’s baseline are critical (e.g., in screening or monitoring programs). In oncology, RI_per_ derived using the PEB framework have enabled earlier tumor detection than traditional fixed cutoffs (7, 11, 23–26). Additionally, studies have shown that hematological biomarkers maintain remarkably stable set points (3), suggesting personalized intervals for such markers can remain valid over decades. Our results indicate that applying PEB thresholds in practice can reduce false-positive flags, potentially minimizing unnecessary follow-up tests.

Despite these advantages, practical challenges exist. Many biomarkers are measured infrequently or only after clinical suspicion arises, which means baseline data for RI_per_ calculations may not be readily available. Careful selection of baseline samples is essential to prevent pathological values from being used as reference points, as this could distort individualized thresholds and misguide clinical interpretation. When prior data are limited or a gradual shift in set point is suspected, evaluating result pairs provides a practical alternative that is more robust to long-term drift. The number of prior results to include should be guided by clinical context, biomarker characteristics, and patient history. Incorporating the PEB algorithm into the LIS for routine reporting can be achieved using BV data published on the EFLM database (27) or estimating from local LIS data using refineR. This approach could assist clinicians in developing on-demand, tailored thresholds suitable for each situation but should be validated in larger clinical studies before implementation.

### Strengths and Limitations

A strength of this study was the use of 2 distinct data sources: routine LIS data and a formal BV study to establish PEB parameters for a broad set of biomarkers with varying BV. We demonstrated that with appropriate data transformations, the PEB approach applies to biomarkers with gaussian or markedly skewed distributions (Supplemental Fig. 26). By offering 2 pathways for parameter derivation, laboratories can potentially extend this approach to underrepresented populations (e.g., pediatric cohorts). Real-world laboratory data inherently captures preanalytical variability and improves the feasibility of applying thresholds in clinical practice. There are, however, acknowledged limitations. LIS-derived parameters may be biased toward pathological values if the source dataset contains a substantial proportion of pathological results, especially if they overlap with nonpathological values. We used refineR and robust regression to mitigate this risk. In practice, we recommend using large datasets (≥5000 samples) with a pathological fraction not exceeding 30%, consistent with established guidance (28). Other potential biases include the failure of the Box-Cox or logarithmic transformations to achieve normality and the presence of autocorrelation in serial measurements. We addressed these by assessing normality (Supplemental Figs. 27 and 28) and requiring at least 24 h between consecutive samples. Sex-specific differences in CV_I_ and CV_G_ may also influence B₁, limiting the generalizability of our steroid results to the male cohort only. We also evaluated the BV parameters on the same dataset from which they were derived. This internal validation could overestimate the performance of the thresholds. Future studies should include female participants and independent external validation cohorts for a more rigorous assessment.

## Conclusion

Our findings demonstrate that the PEB framework effectively provides personalized cutoffs. The method yields narrower reference limits compared to population-based reference intervals while maintaining the intended proportion of flagged results across serial measurements. We showed that the required PEB parameters can be derived from BV or routine LIS data, highlighting 2 practical pathways for clinical implementation. A PEB approach could enhance diagnostic accuracy by tailoring thresholds to individual patients.

## Supplementary Material

hvaf092_Supplementary_Data

## Data Availability

All data is available from the corresponding author upon reasonable request.

## Nonstandard Abbreviations

RI_pop_, population reference intervals; RI_per_, personal reference intervals; CV_I_, within-subject biological variation; CV_G_, between-subject biological variation; RCV, reference change values; PEB, parametric empirical Bayes; BV, biological variation; LIS, laboratory information system; CV_A_, analytical variation; 11-DOC, 11-deoxycortisol; 17-OHP, 17-hydroxyprogesterone.
