# Supplementary material for: A Parametric Empirical Bayes Approach to Personalized Reference Intervals and Reference Change Values
Source: Clin Chem. 2025 Aug 22;71(11):1147–57. doi: 10.1093/clinchem/hvaf092 (PMC12582661; doi:10.1093/clinchem/hvaf092)
Supplement: hvaf092_Supplementary_Data [file hvaf092_supplementary_data.zip › ClinChem-2025-0318.R4_Supplemental_Tables_and_Figures_Final.docx]

**Supplemental Tables and Figure**

**Suppl. Table 1:** Lower reporting limits for the biomarkers in the study

| **Measurand** | **Lower Reporting Limit** |
| --- | --- |
| Albumin | 0.2 g/dL |
| Creatinine^a^ | 0.06 mg/dL |
| Phosphate^a^ | 0.30 mg/dL |
| Cortisone^a^ | 0.023 µg/dL |
| Cortisol^a^ | 0.18 µg/dL |
| Testosterone^a^ | 2.9 ng/dL |
| Androstenedione^a^ | 5.7 ng/dL |
| 17-Hydroxyprogestrone^a^ | 9.9 ng/dL |
| 11-Deoxycortisol^a^ | 10.4 ng/dL |

^a:^The original measurements were in moles and then converted to conventional units using the following conversion factors: creatinine: 1 µmol/L = 0.0113 mg/dL, phosphate: 1 mmol/L = 3.1 mg/dL, cortisone: 1 nmol/L = 0.0360 µg/dL, cortisol: 1 nmol/L = 0.0362 µg/dL, testosterone: 1 nmol/L = 28.8 ng/dL, androstenedione: 1 nmol/L = 28.6 ng/dL, 17-hydroxyprogesterone: 1 nmol/L = 33 ng/dL, 11-deoxycortisol: 1 nmol/L = 34.7 ng/dL


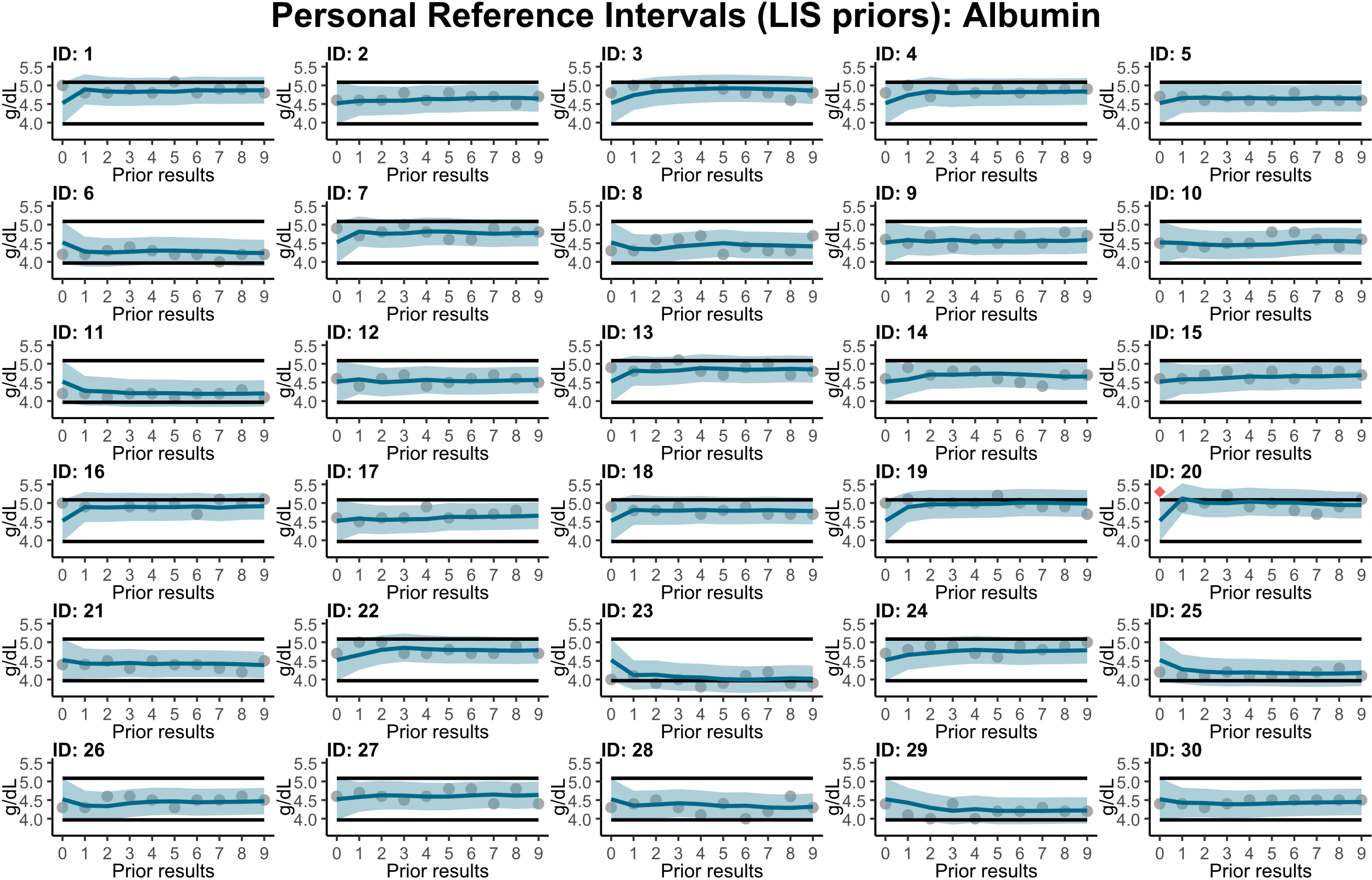

**Suppl. Figure 1:** Personalized reference intervals (RI_per_) for albumin, with a two-sided 95% prediction interval (shaded blue area), using PEB parameters based on laboratory information system data, across measurements from male participants (dots), in the biological variation study. Horizontal black lines represent the 95% reference interval determined by the refineR algorithm, while the red diamond denotes a flagged measurement exceeding the RI_per_.

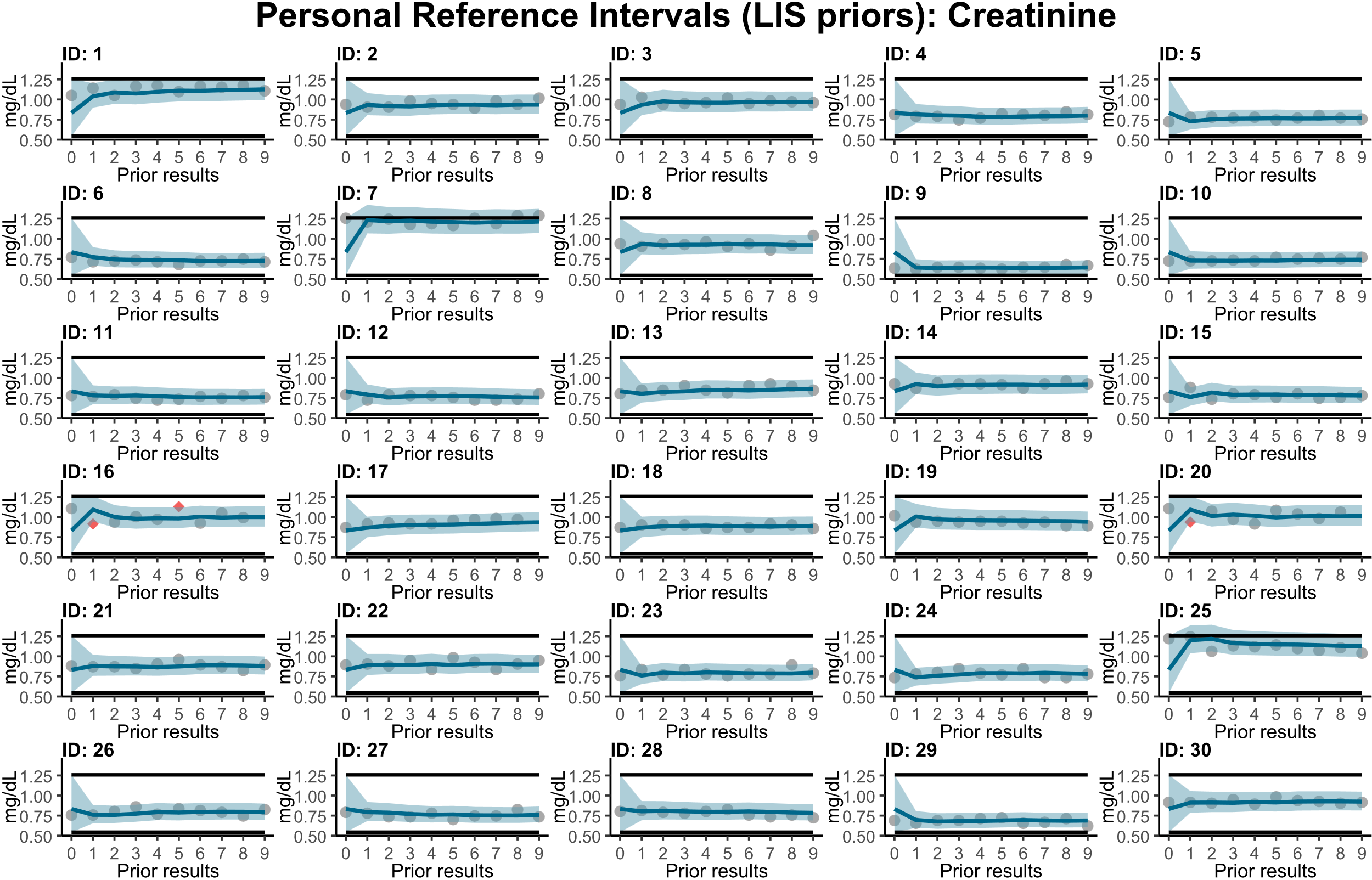

**Suppl. Figure 2**: Personalized reference intervals for creatinine (RI_per_), with a two-sided 95% prediction interval (shaded blue area), using PEB parameters based on laboratory information system data, across measurements from male participants (dots) in the biological variation study. Horizontal black lines represent the 95% reference interval determined by the refineR algorithm, while the red diamond denotes a flagged measurement exceeding the RI_per_.

**Personalized Reference Intervals (LIS-parameters): Albumin**

**Personalized Reference Intervals (LIS-parameters): Creatinine**


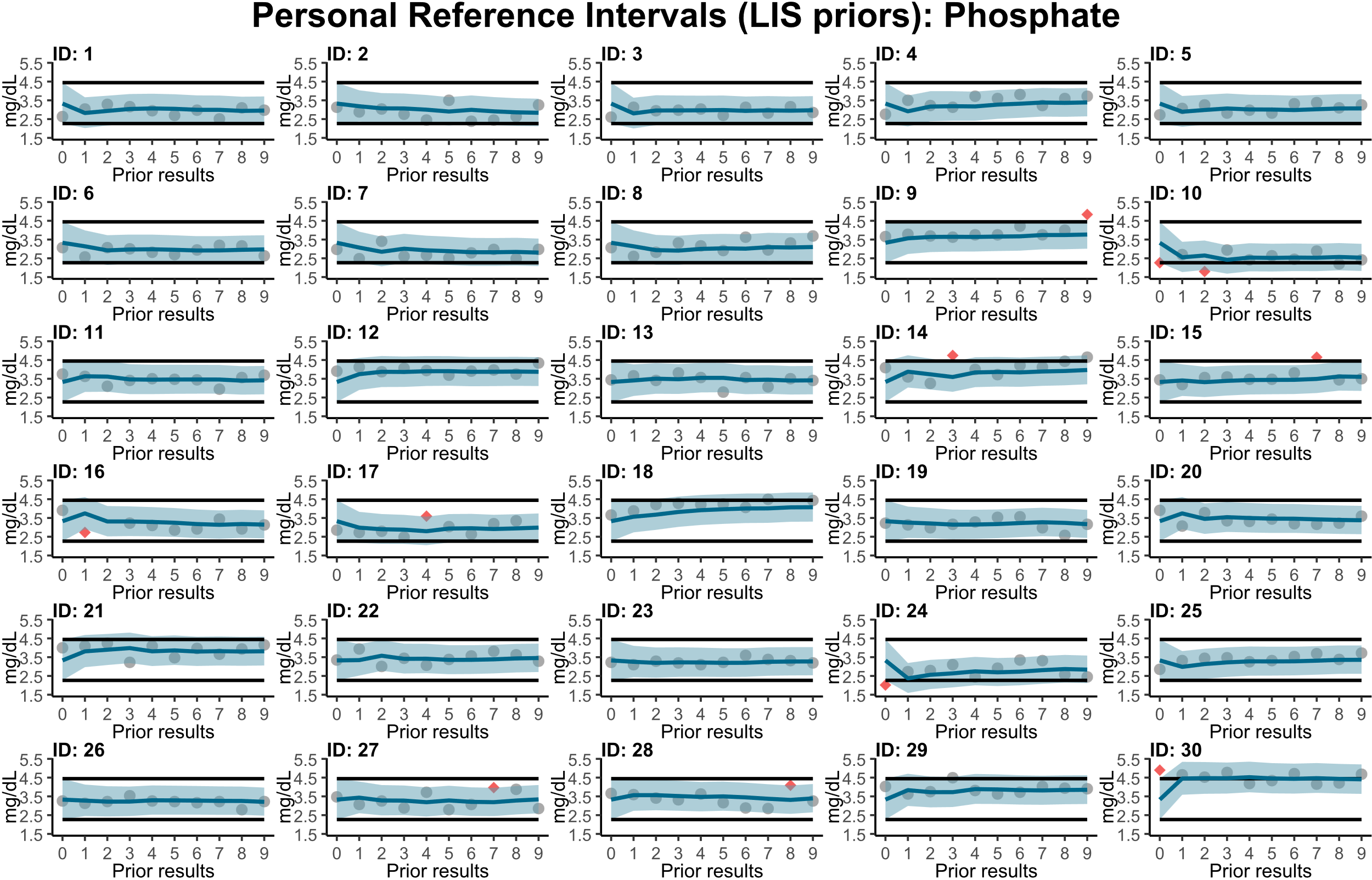

**Suppl. Figure 3:** Personalized reference intervals for phosphate (RI_per_) (shaded blue area), with a two-sided 95% prediction interval,with PEB parameters based on laboratory information system data, across measurements from male participants (dots) in the biological variation study. Horizontal black lines represent the 95% reference interval determined by the refineR algorithm, while the red diamond denotes a flagged measurement exceeding the RI_per_.

**Personalized Reference Intervals (LIS-parameters): Phosphate**

**Personalized Reference Intervals (LIS-parameters): Cortisone**


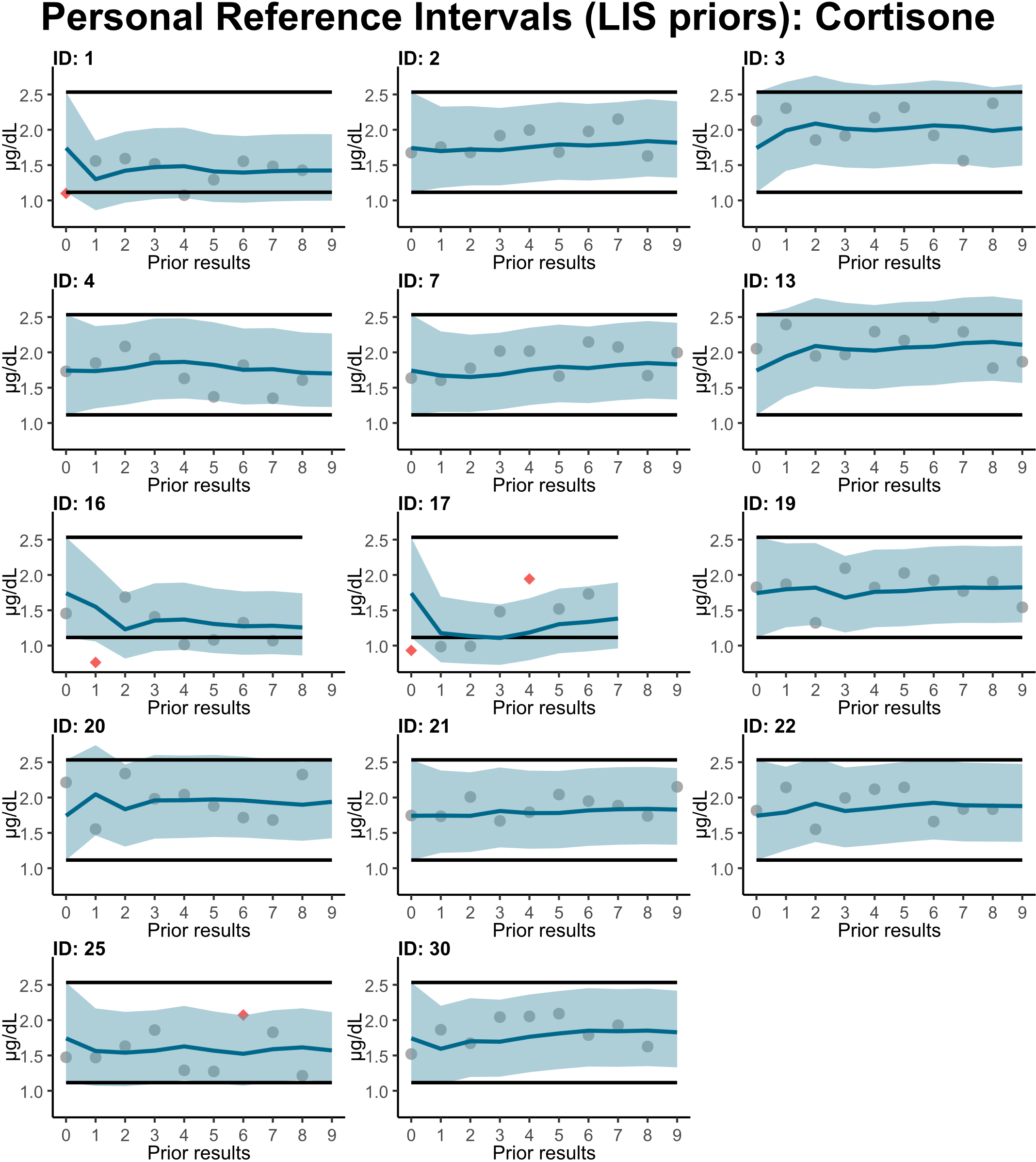


**Suppl. Figure 4**: Personalized reference intervals for cortisone (RI_per_), with a two-sided 95% prediction interval (shaded blue area), using PEB parameters based on laboratory information system data, across measurements from male participants (dots) in the biological variation study. Horizontal black lines represent the 95% reference interval determined by the refineR algorithm, while the red diamond denotes a flagged measurement exceeding the RI_per_.

**Personalized Reference Intervals (LIS-parameters): Cortisol**


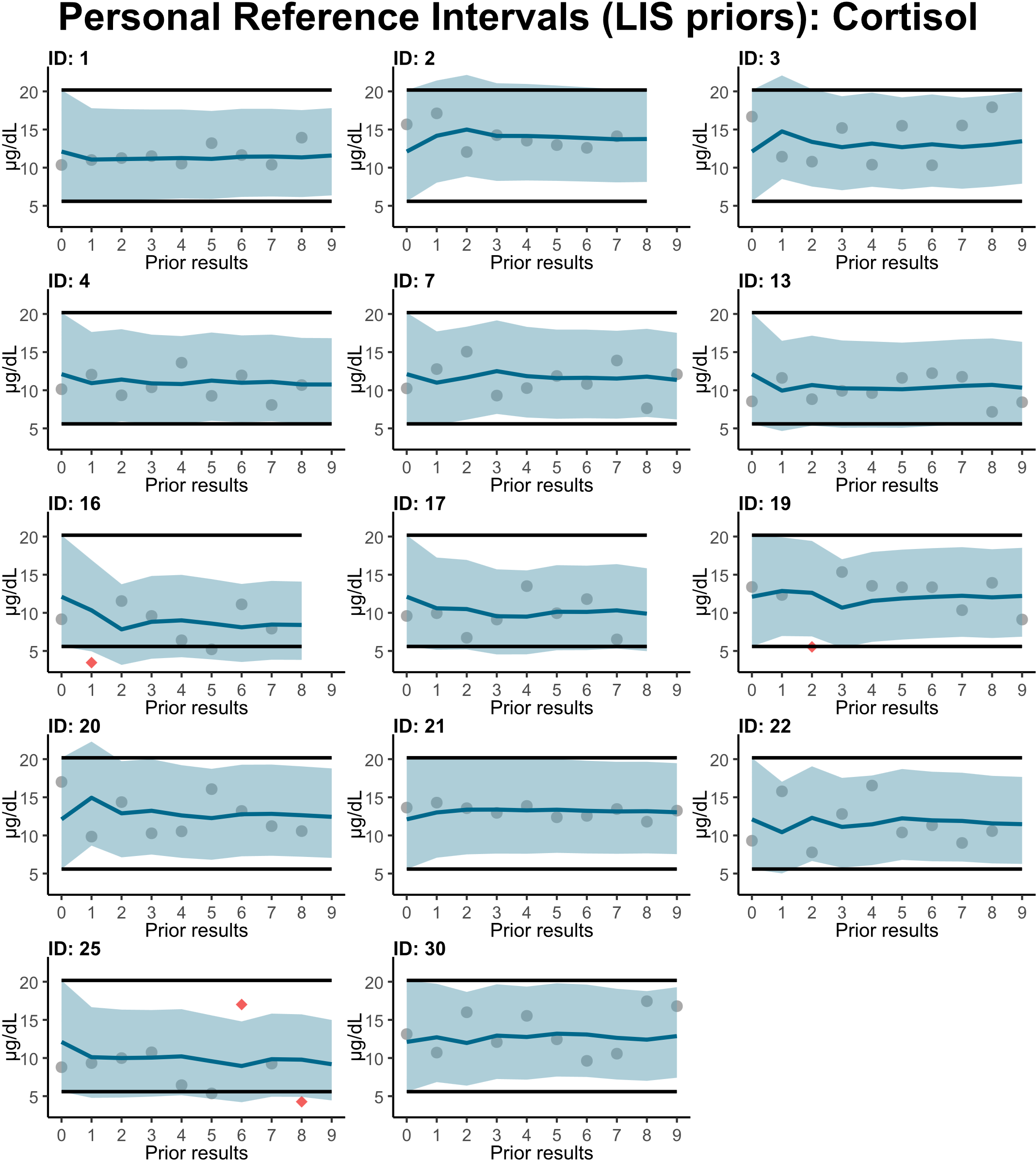


**Suppl. Figure 5:** Personalized reference intervals for cortisol (RI_per_), with a two-sided 95% prediction interval (shaded blue area), using PEB parameters based on laboratory information system data, across measurements from male participants (dots) Horizontal black lines represent the 95% reference interval determined by the refineR algorithm, while the red diamond denotes a flagged measurement exceeding the RI_per_.
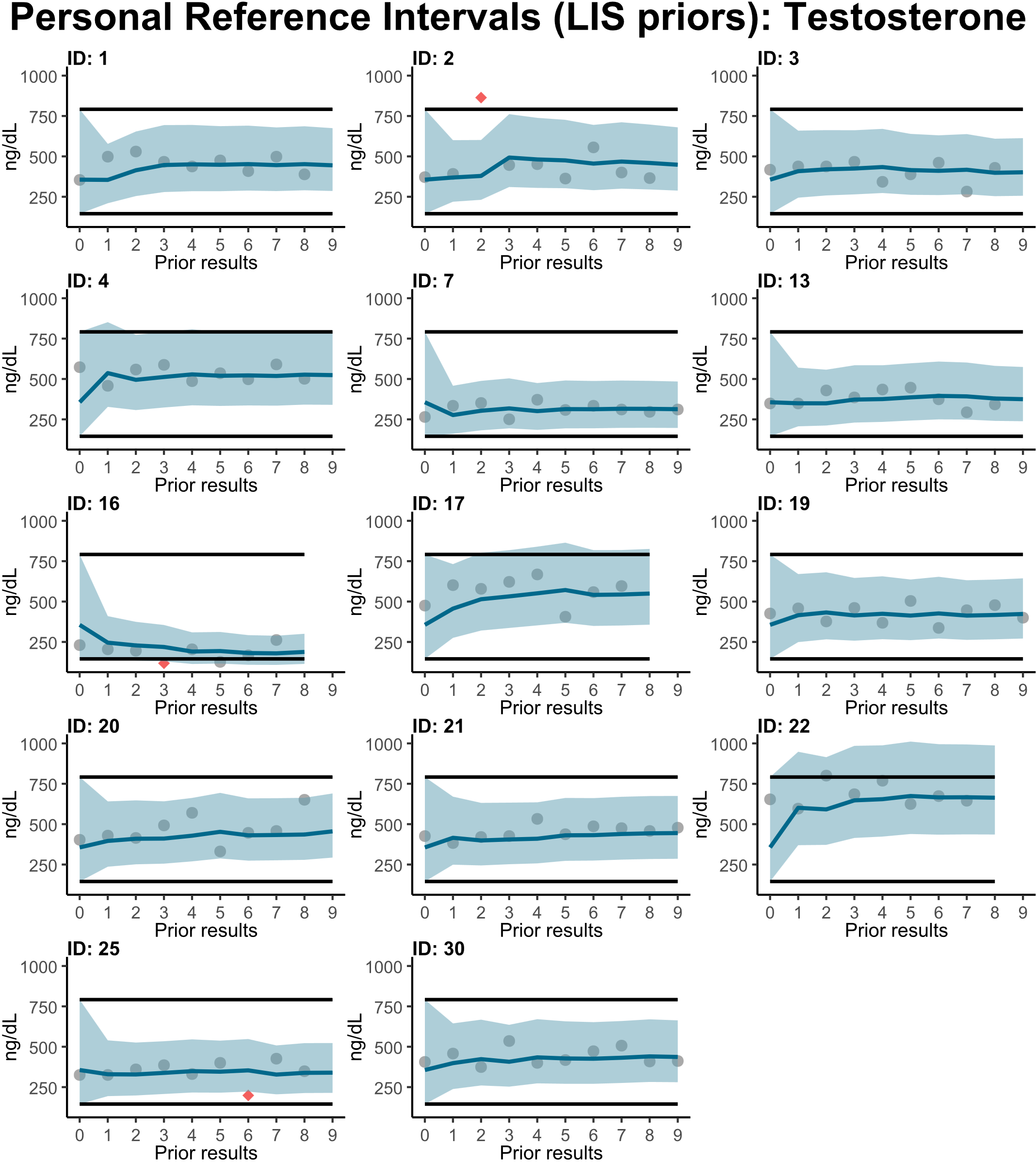


**Personalized Reference Intervals (LIS-parameters): Testosterone**

**Suppl. Figure 6:** Personalized reference intervals for testosterone (RI_per_), with a two-sided 95% prediction interval (shaded blue area), using PEB parameters based on laboratory information system data, across measurements from male participants (dots) Horizontal black lines represent the 95% reference interval determined by the refineR algorithm, while the red diamond denotes a flagged measurement exceeding the RI_per_.


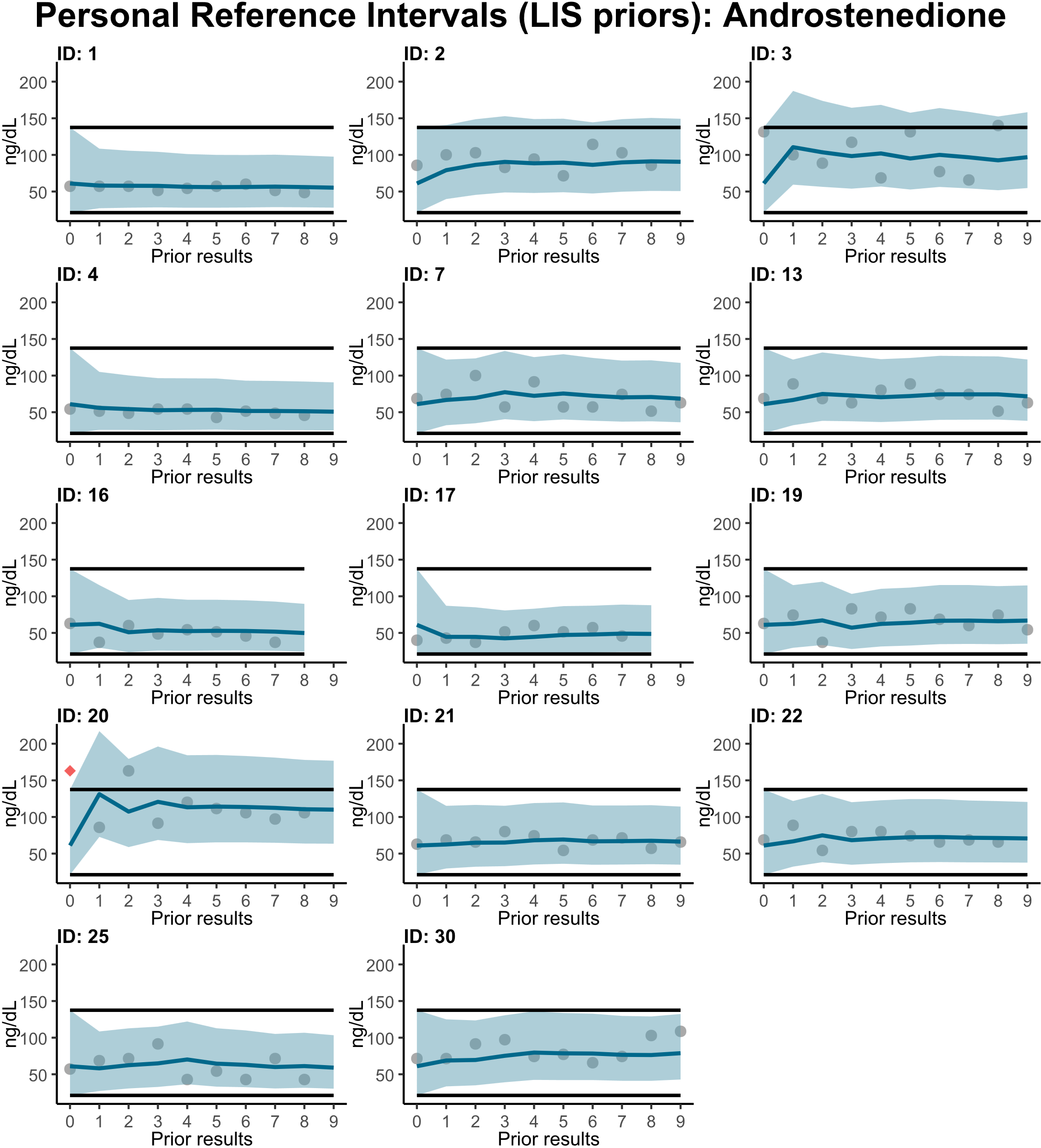


**Personalized Reference Intervals (LIS-parameters): Androstenedione**

**Suppl. Figure 7:** Personalized reference intervals for androstenedione (RI_per_), with a two-sided 95% prediction interval (shaded blue area), using PEB parameters based on laboratory information system data, across measurements from male participants (dots) in the biological variation study. Horizontal black lines represent the 95% reference interval determined by the refineR algorithm, while the red diamond denotes a flagged measurement exceeding the RI_per_.


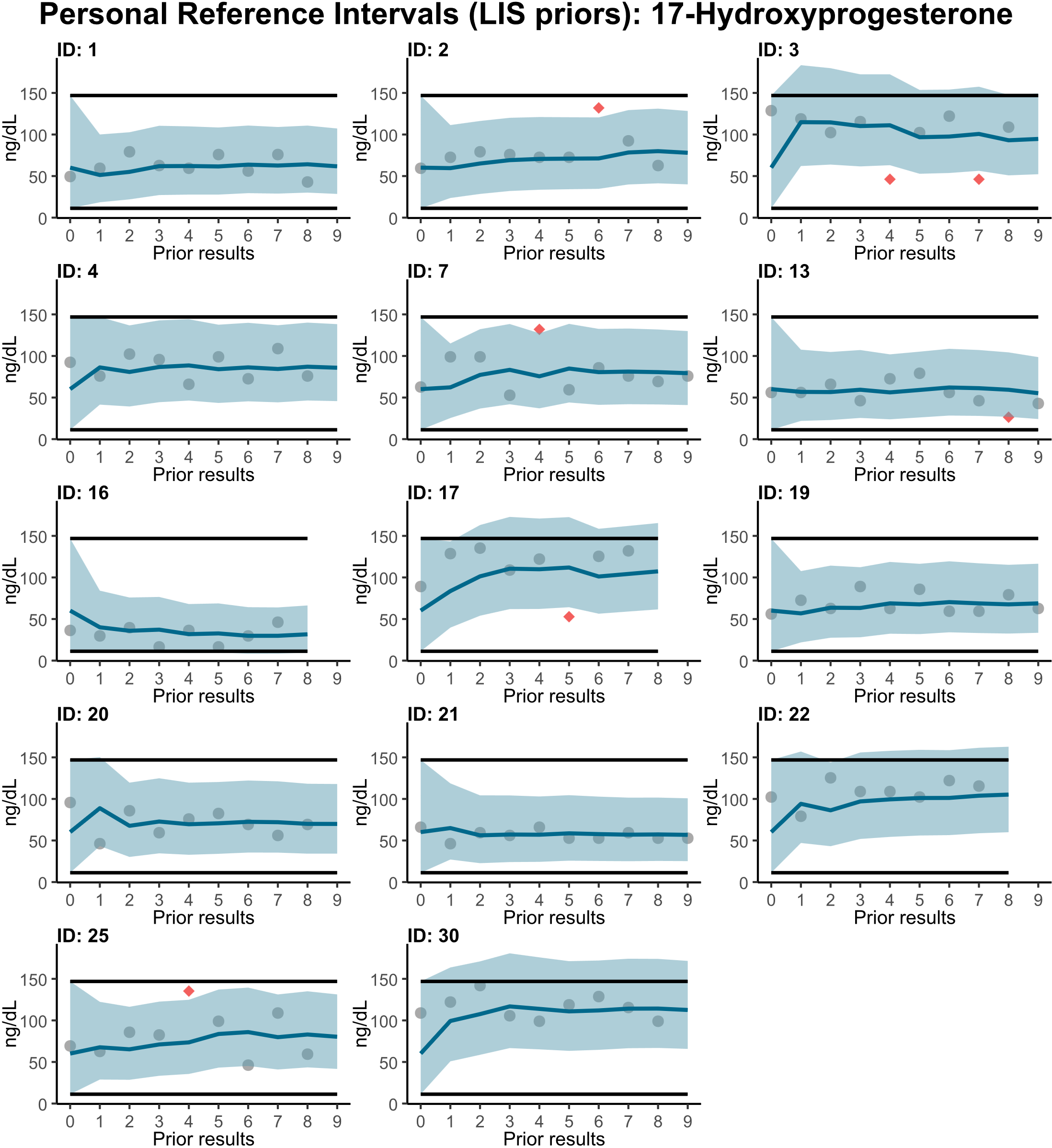


**Personalized Reference Intervals (LIS-parameters): 17-Hydroxyprogesterone**

**Suppl. Figure 8:** Personalized reference intervals for 17-Hydroxyprogesterone (RI_per_), with a two-sided 95% prediction interval (shaded blue area), using PEB parameters based on laboratory information system data, across measurements from male participants (dots) in the biological variation study. Horizontal black lines represent the 95% reference interval determined by the refineR algorithm, while the red diamond denotes a flagged measurement exceeding the RI_per_.


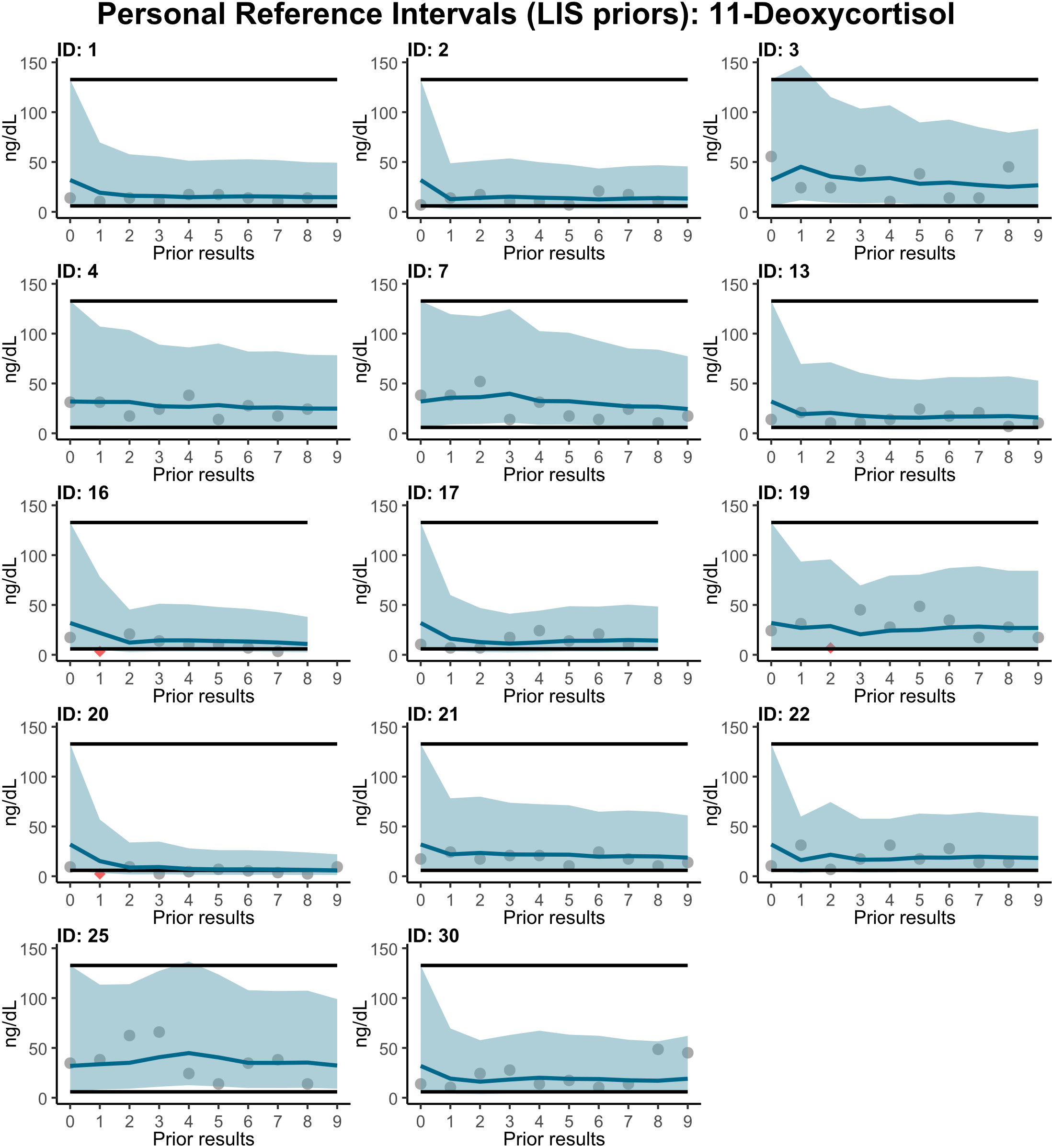

**Suppl. Figure 9**: Personalized reference intervals for 11-Deoxycortisol (RI_per_), with a two-sided 95% prediction interval (shaded blue area), using PEB parameters based on laboratory information system data, across measurements from male participants (dots) in the biological variation study. Horizontal black lines represent the 95% reference interval determined by the refineR algorithm, while the red diamond denotes a flagged measurement exceeding the RI_per_.

**Personalized Reference Intervals (LIS-parameters): 11-Deoxycortisol**

**Suppl.
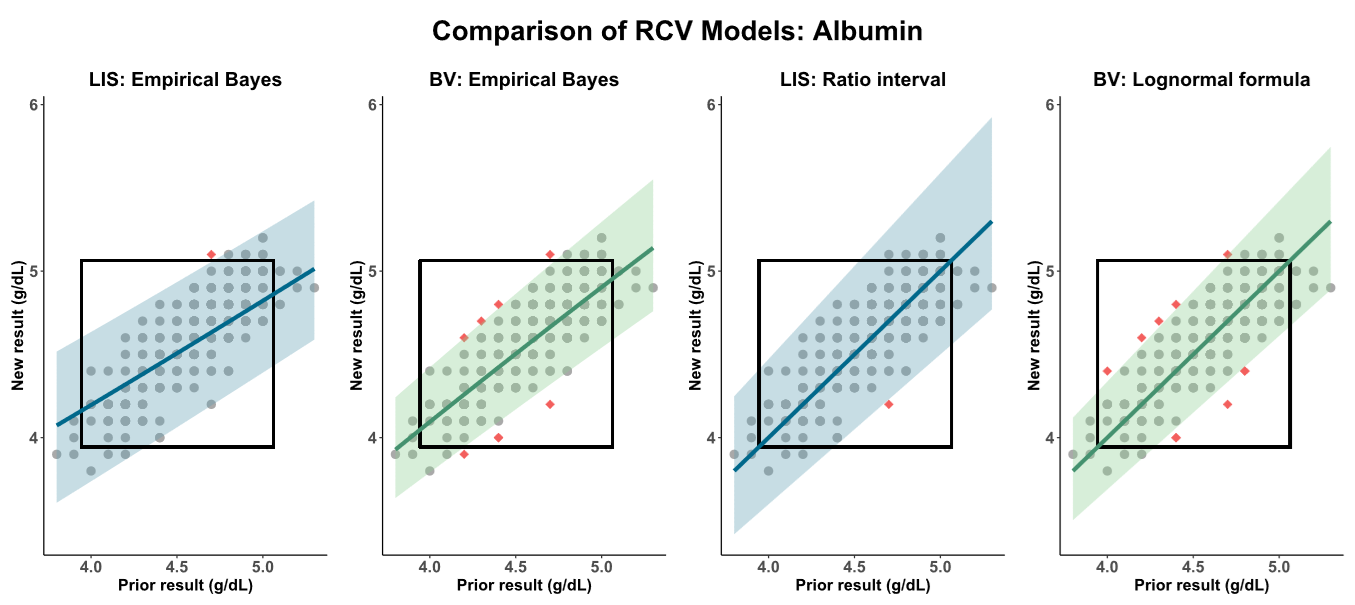
Figure 10:** Reference change values (RCVs) with a two-sided 95% prediction interval (shaded area) for albumin, compared to result pairs (dots) from serial measurements of participants in the biological variation study. The four panels compare different RCV models: (1) Parametric Empirical Bayes with laboratory information system (LIS) parameters, (2) Parametric Empirical Bayes with parameters based on biological variation (BV) estimates, (3) Ratio interval distributions derived from LIS data, and (4) a BV-based lognormal formula. Red diamonds indicate flagged result pairs exceeding the RCV thresholds. The central black square is the reference interval estimated by refineR for visual comparison.


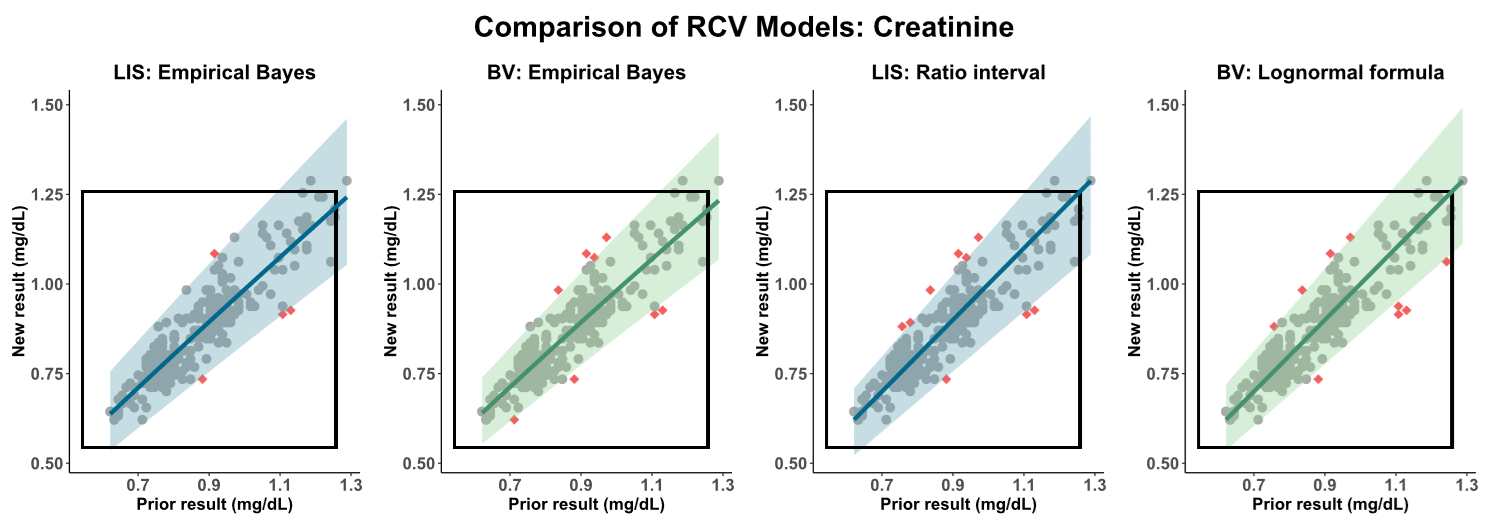


**Suppl. Figure 11:** Reference change values (RCVs) with a two-sided 95% prediction interval (shaded area) for creatinine, compared to result pairs (dots) from serial measurements of participants in the biological variation study. The four panels compare different RCV models: (1) Parametric Empirical Bayes with laboratory information system (LIS) parameters, (2) Parametric Empirical Bayes with parameters based on biological variation (BV) estimates, (3) Ratio interval distributions derived from LIS data, and (4) a BV-based lognormal formula. Red diamonds indicate flagged result pairs exceeding the RCV thresholds. The central black square is the reference interval estimated by refineR for visual comparison.
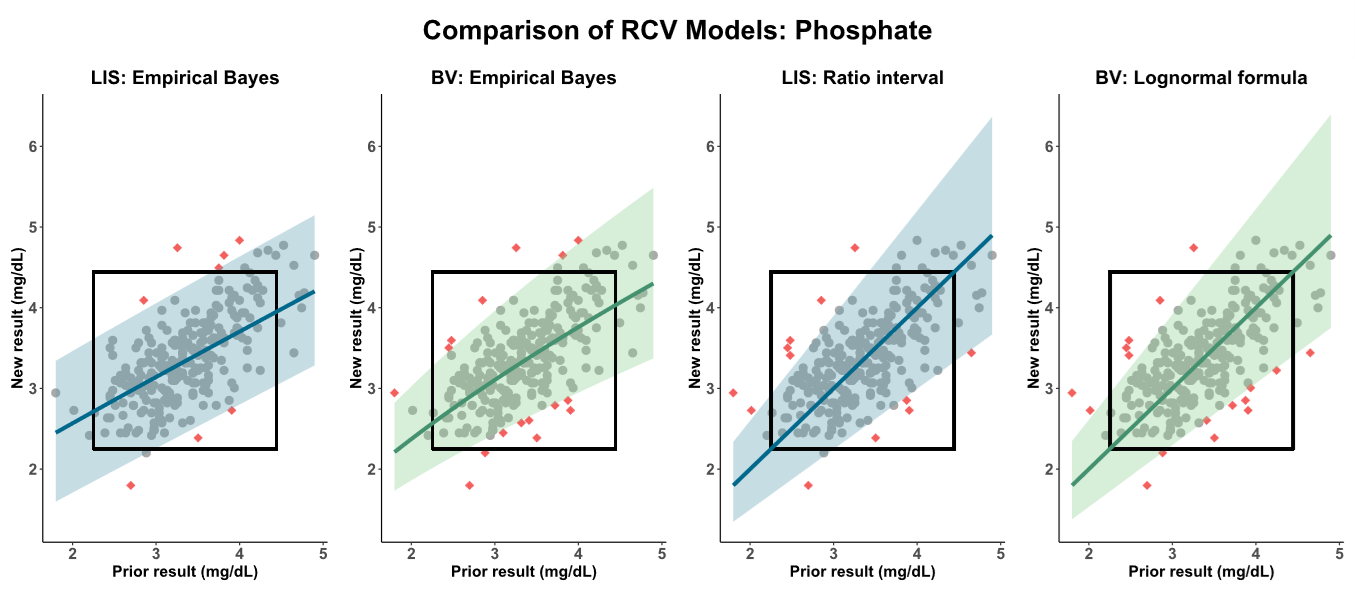

**Suppl. Figure 12:** Reference change values (RCVs) with a two-sided 95% prediction interval (shaded area) for phosphate, compared to result pairs (dots) from serial measurements of participants in the biological variation study. The four panels compare different RCV models: (1) Parametric Empirical Bayes with laboratory information system (LIS) parameters, (2) Parametric Empirical Bayes with parameters based on biological variation (BV) estimates, (3) Ratio interval distributions derived from LIS data, and (4) a BV-based lognormal formula. Red diamonds indicate flagged result pairs exceeding the RCV thresholds. The central black square is the reference interval estimated by refineR for visual comparison.
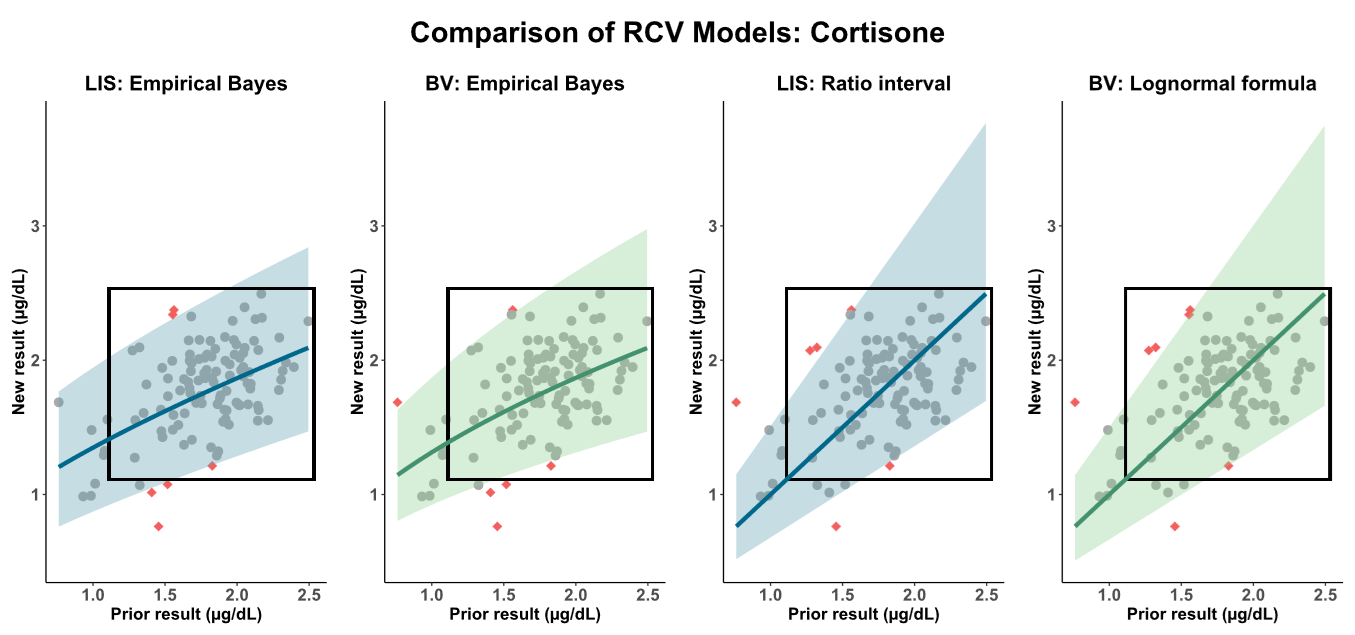

**Suppl. Figure 13:** Reference change values (RCVs) with a two-sided 95% prediction interval (shaded area) for cortisone, compared to result pairs (dots) from serial measurements of male participants in the biological variation study. The four panels compare different RCV models: (1) Parametric Empirical Bayes with laboratory information system (LIS) parameters, (2) Parametric Empirical Bayes with parameters based on biological variation (BV) estimates, (3) Ratio interval distributions derived from LIS data, and (4) a BV-based lognormal formula. Red diamonds indicate flagged result pairs exceeding the RCV thresholds. The central black square is the reference interval estimated by refineR for visual comparison.


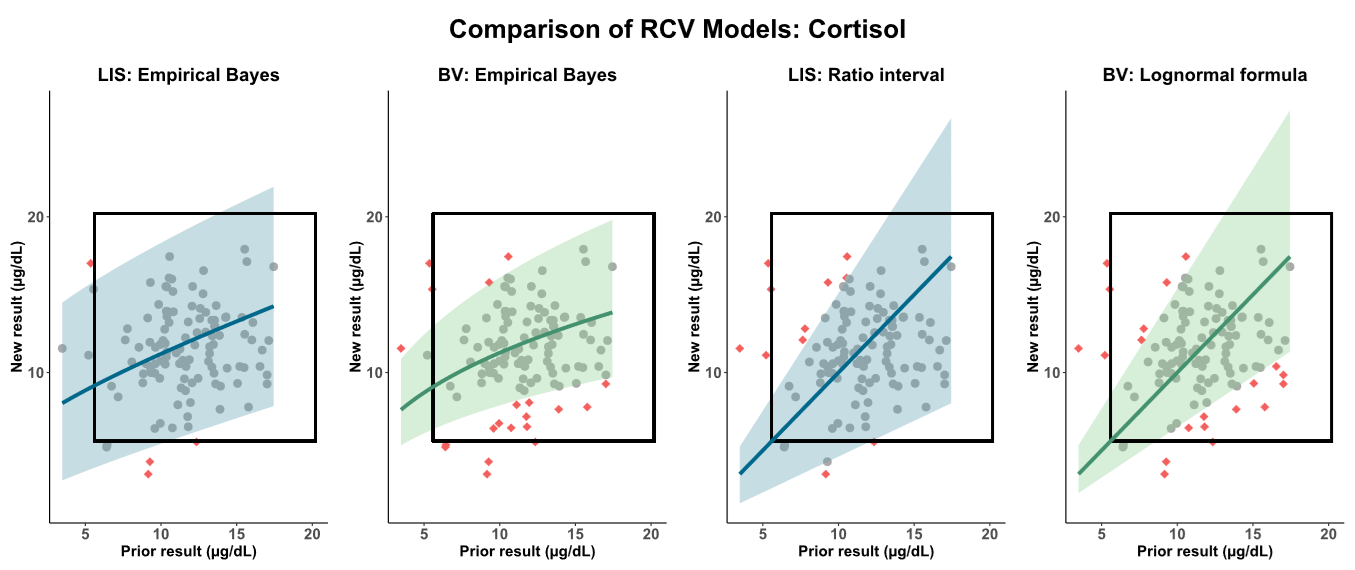


**Suppl. Figure 14:** Reference change values (RCVs) with a two-sided 95% prediction interval (shaded area) for cortisol, compared to result pairs (dots) from serial measurements of male participants in the biological variation study. The four panels compare different RCV models: (1) Parametric Empirical Bayes with laboratory information system (LIS) parameters, (2) Parametric Empirical Bayes with parameters based on biological variation (BV) estimates, (3) Ratio interval distributions derived from LIS data, and (4) a BV-based lognormal formula. Red diamonds indicate flagged result pairs exceeding the RCV thresholds. The central black square is the reference interval estimated by refineR for visual comparison.
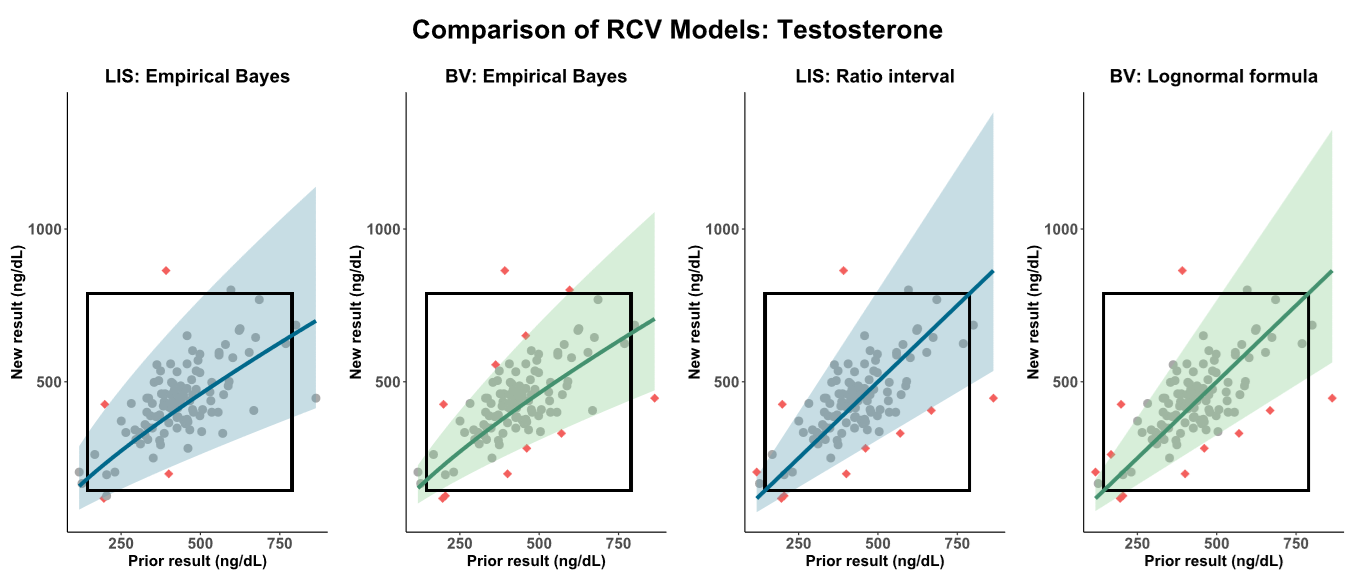


**Suppl. Figure 15:** Reference change values (RCVs) with a two-sided 95% prediction interval (shaded area) for testosterone, compared to result pairs (dots) from serial measurements of male participants in the biological variation study. The four panels compare different RCV models: (1) Parametric Empirical Bayes with laboratory information system (LIS) parameters, (2) Parametric Empirical Bayes with parameters based on biological variation (BV) estimates, (3) Ratio interval distributions derived from LIS data, and (4) a BV-based lognormal formula. Red diamonds indicate flagged result pairs exceeding the RCV thresholds. The central black square is the reference interval estimated by refineR for visual comparison.


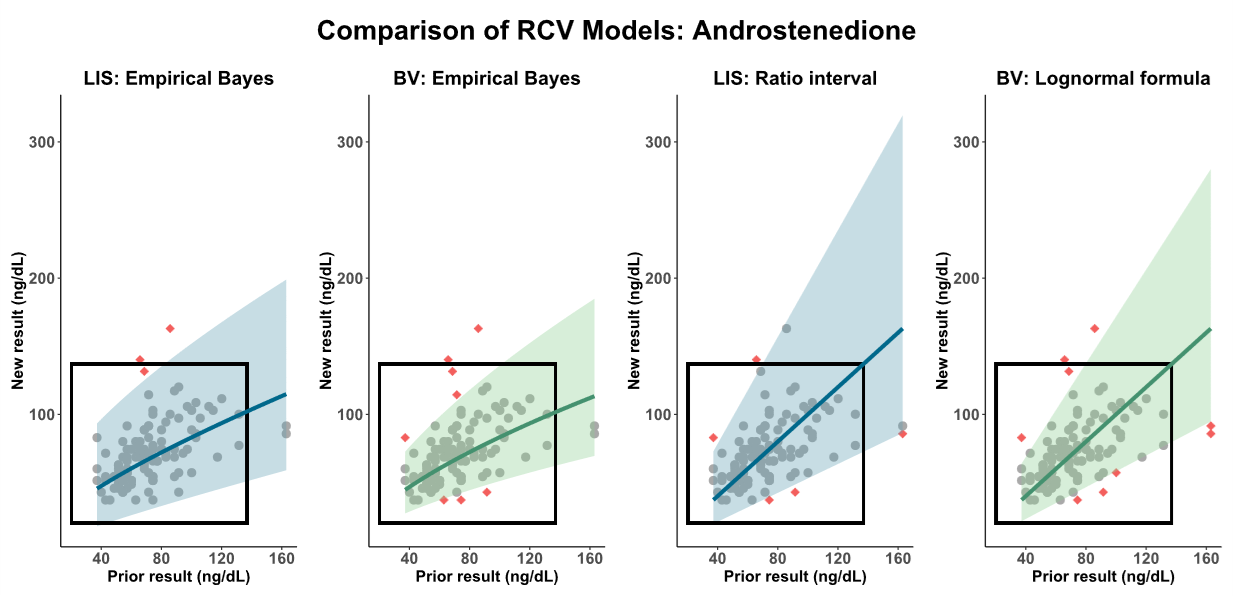


**Suppl. Figure 16:** Reference change values (RCVs) with a two-sided 95% prediction interval (shaded area) for adrostenedione, compared to result pairs (dots) from serial measurements of male participants in the biological variation study. The four panels compare different RCV models: (1) Parametric Empirical Bayes with laboratory information system (LIS) parameters, (2) Parametric Empirical Bayes with parameters based on biological variation (BV) estimates, (3) Ratio interval distributions derived from LIS data, and (4) a BV-based lognormal formula. Red diamonds indicate flagged result pairs exceeding the RCV thresholds. The central black square is the reference interval estimated by refineR for visual comparison.

**
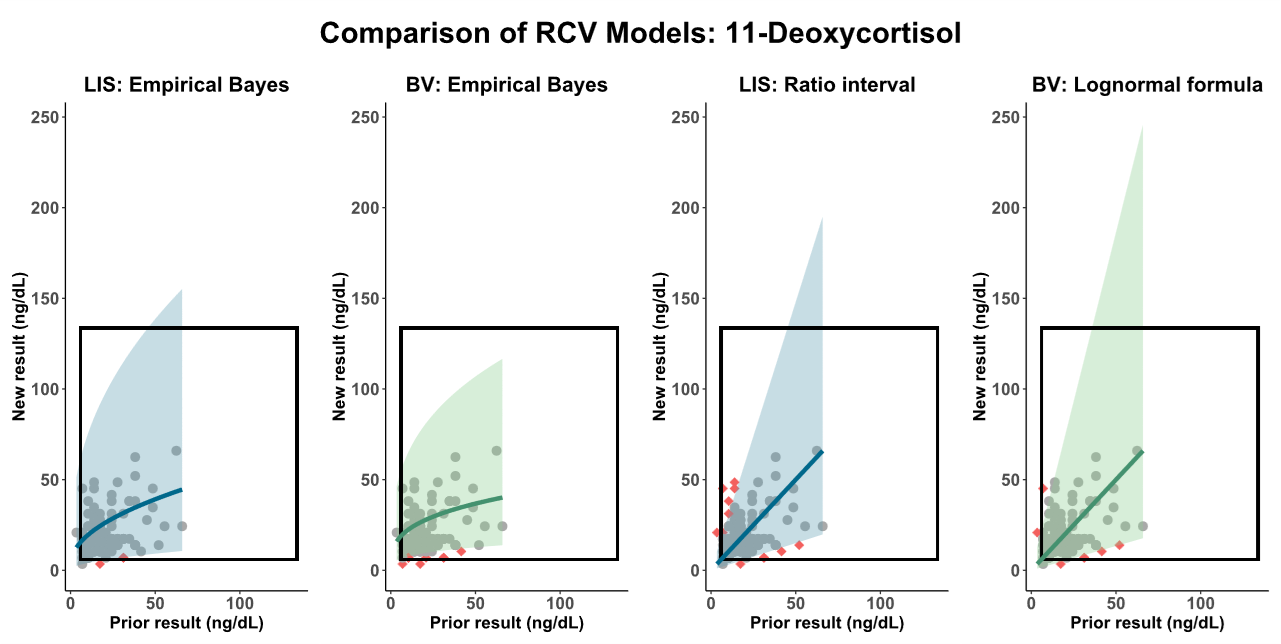

Suppl. Figure 17:** Reference change values (RCVs) with a two-sided 95% prediction interval (shaded area) for 11-Deoxycortisol, compared to result pairs (dots) from serial measurements of male participants in the biological variation study. The four panels compare different RCV models: (1) Parametric Empirical Bayes with laboratory information system (LIS) parameters, (2) Parametric Empirical Bayes with parameters based on biological variation (BV) estimates, (3) Ratio interval distributions derived from LIS data, and (4) a BV-based lognormal formula. Red diamonds indicate flagged result pairs exceeding the RCV thresholds. The central sblack square is the reference interval estimated by refineR for visual comparison.

**
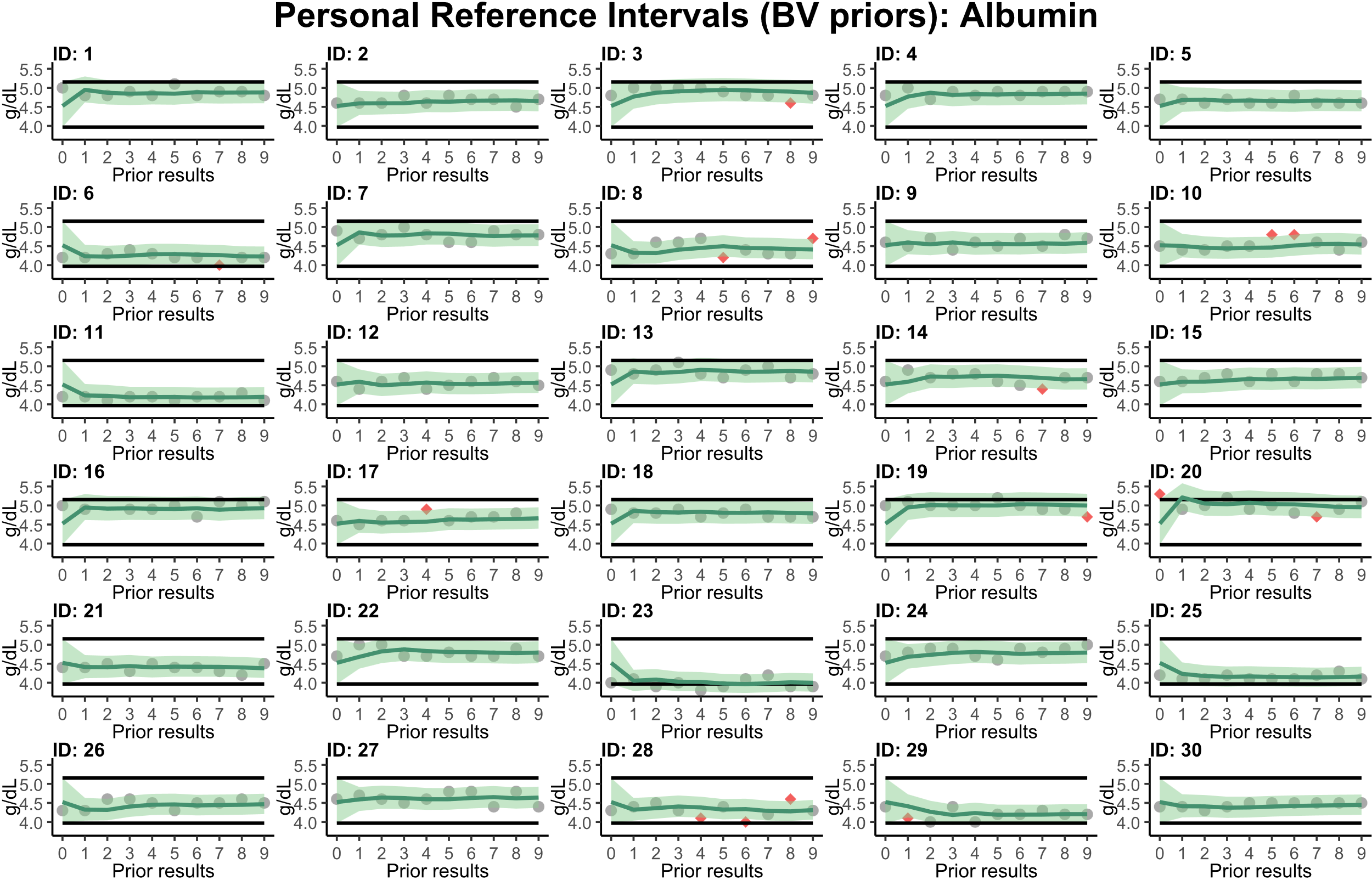
 Suppl. Figure 18:** Personalized reference intervals for albumin (RI_per_), with a two-sided 95% prediction interval (shaded green area), using PEB parameters based on biological variation estimates, across measurements from all participants (dots) in the biological variation study. Horizontal black lines represent the 95% reference interval determined by the refineR algorithm, while the red diamond denotes a flagged measurement exceeding the RI_per_.

**Personalized Reference Intervals (BV-parameters): Albumin**

**Personalized Reference Intervals (BV-parameters): Creatinine**


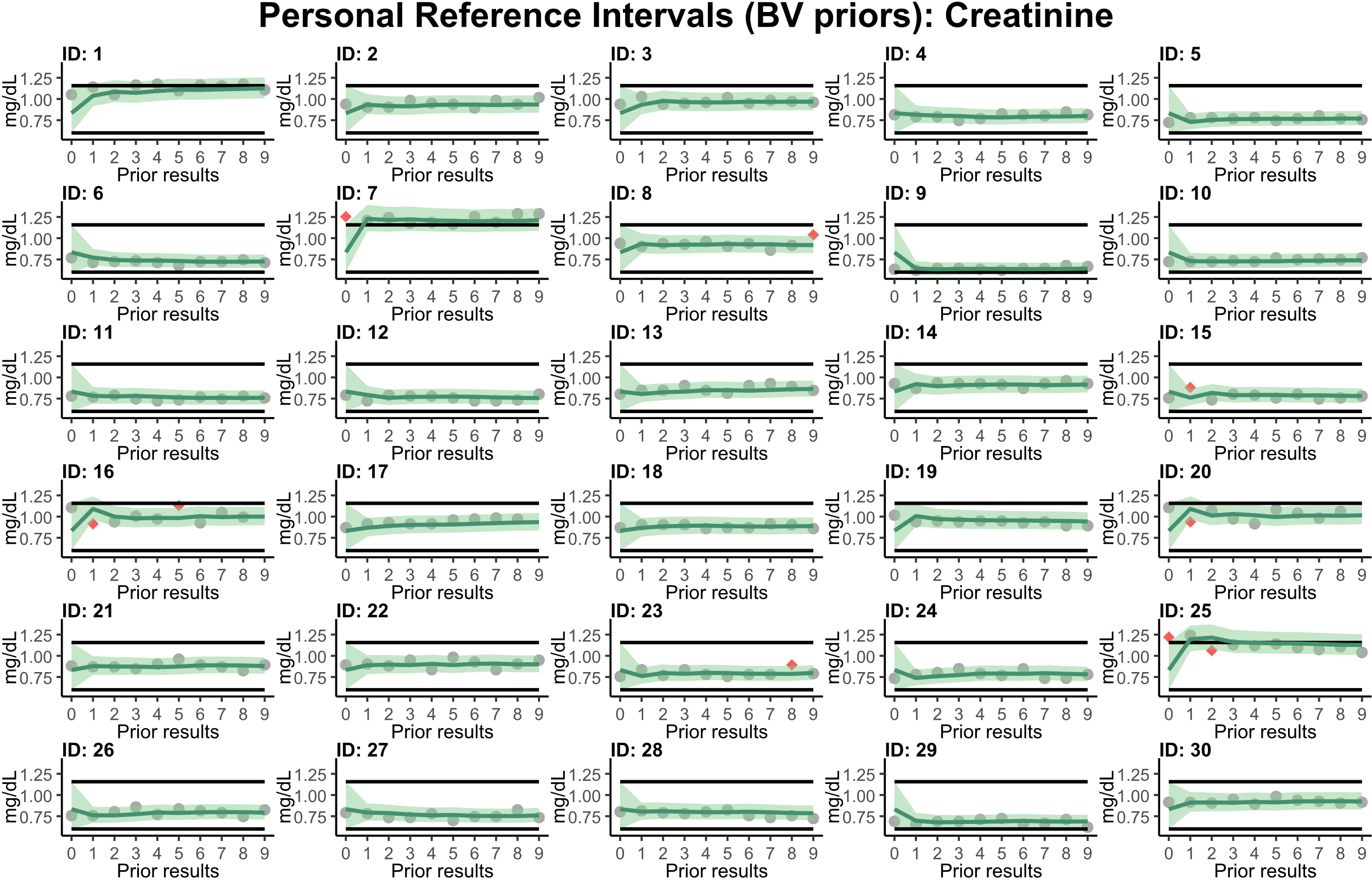
 **Suppl. Figure 19:** Personalized reference intervals for creatinine (RI_per_), with a two-sided 95% prediction interval (shaded green area), using PEB parameters based on biological variation estimates, across measurements from all participants (dots) in the biological variation study. Horizontal black lines represent the 95% reference interval determined by the refineR algorithm, while the red diamond denotes a flagged measurement exceeding the RI_per_.


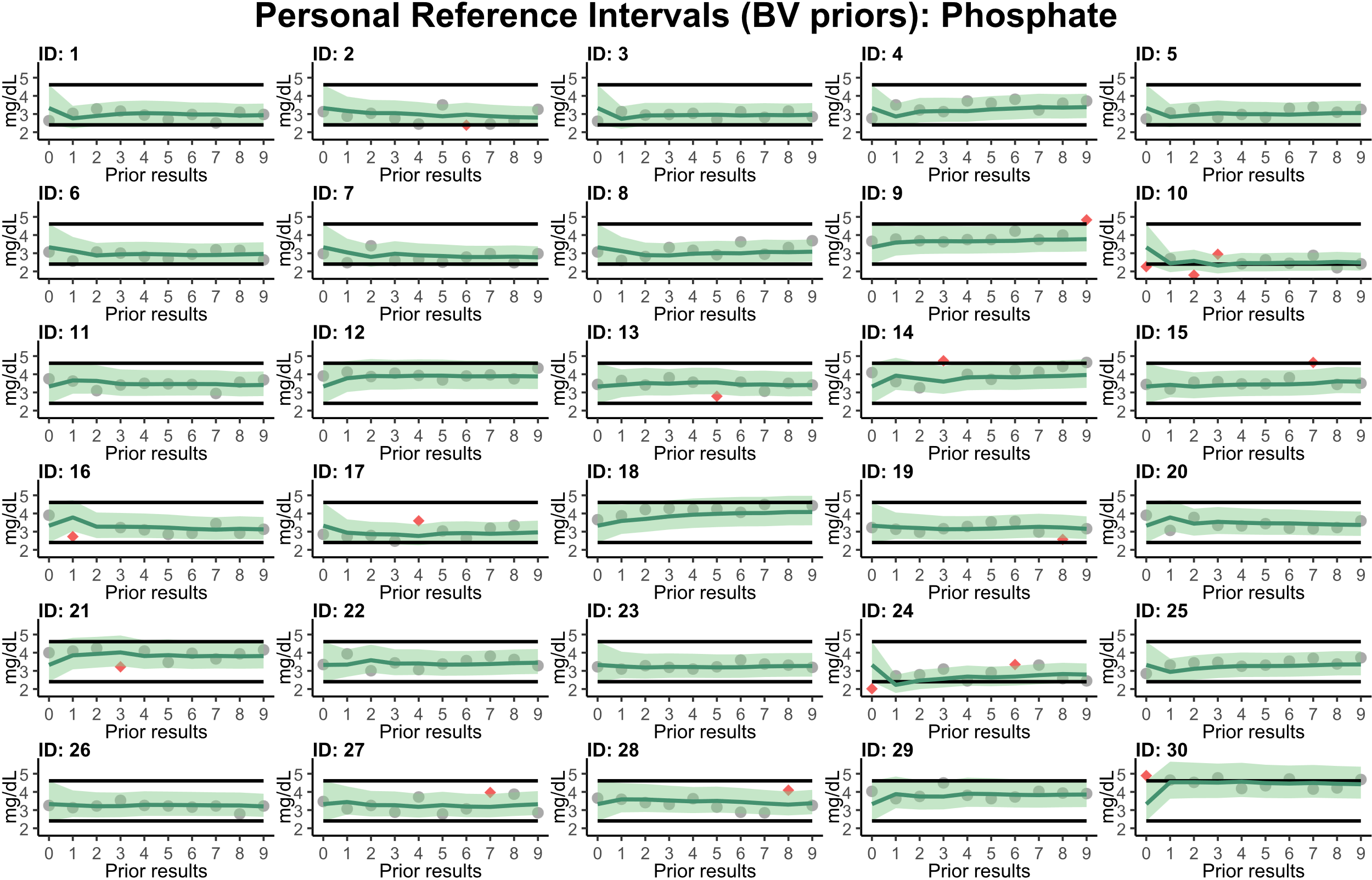
 **Suppl. Figure 20:** Personalized reference intervals for phosphate (RI_per_), with a two-sided 95% prediction interval (shaded green area), using PEB parameters based on biological variation estimates, across measurements from all participants (dots) in the biological variation study. Horizontal black lines represent the 95% reference interval determined by the refineR algorithm, while the red diamond denotes a flagged measurement exceeding the RI_per_.

**Personalized Reference Intervals (BV-parameters): Phosphate**

**Personalized Reference Intervals (BV-parameters): Cortisone**


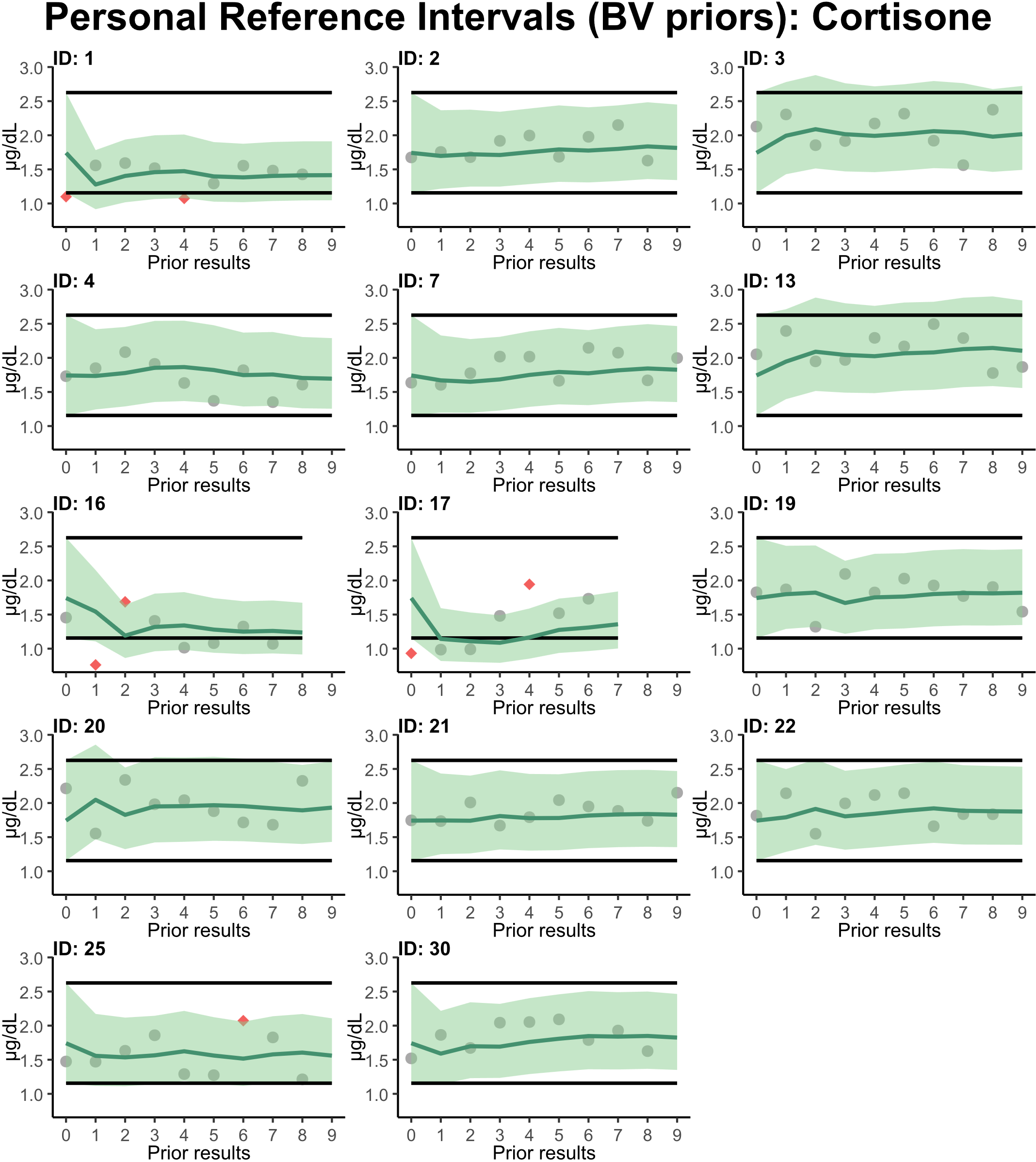


**Suppl. Figure 21:** Personalized reference intervals for cortisone (RI_per_), with a two-sided 95% prediction interval (shaded green area), using PEB parameters based on biological variation estimates, across measurements from male participants (dots) in the biological variation study. Horizontal black lines represent the 95% reference interval determined by the refineR algorithm, while the red diamond denotes a flagged measurement exceeding the RI_per_.
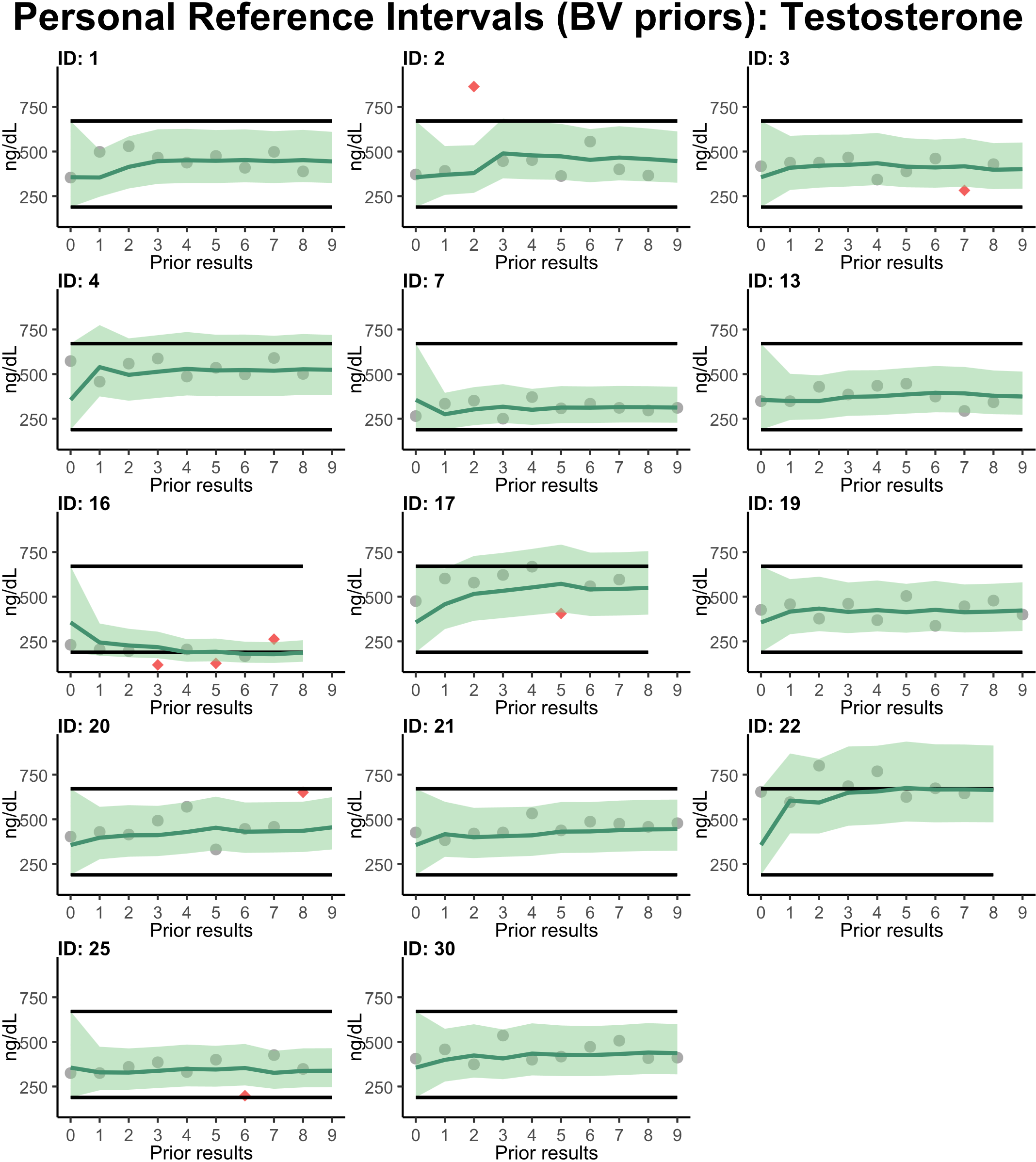

**Suppl. Figure 22:** Personalized reference intervals for testosterone (RI_per_), with a two-sided 95% prediction interval (shaded green area), using PEB parameters based on biological variation estimates, across measurements from male participants (dots) in the biological variation study. Horizontal black lines represent the 95% reference interval determined by the refineR algorithm, while the red diamond denotes a flagged measurement exceeding the RI_per_.
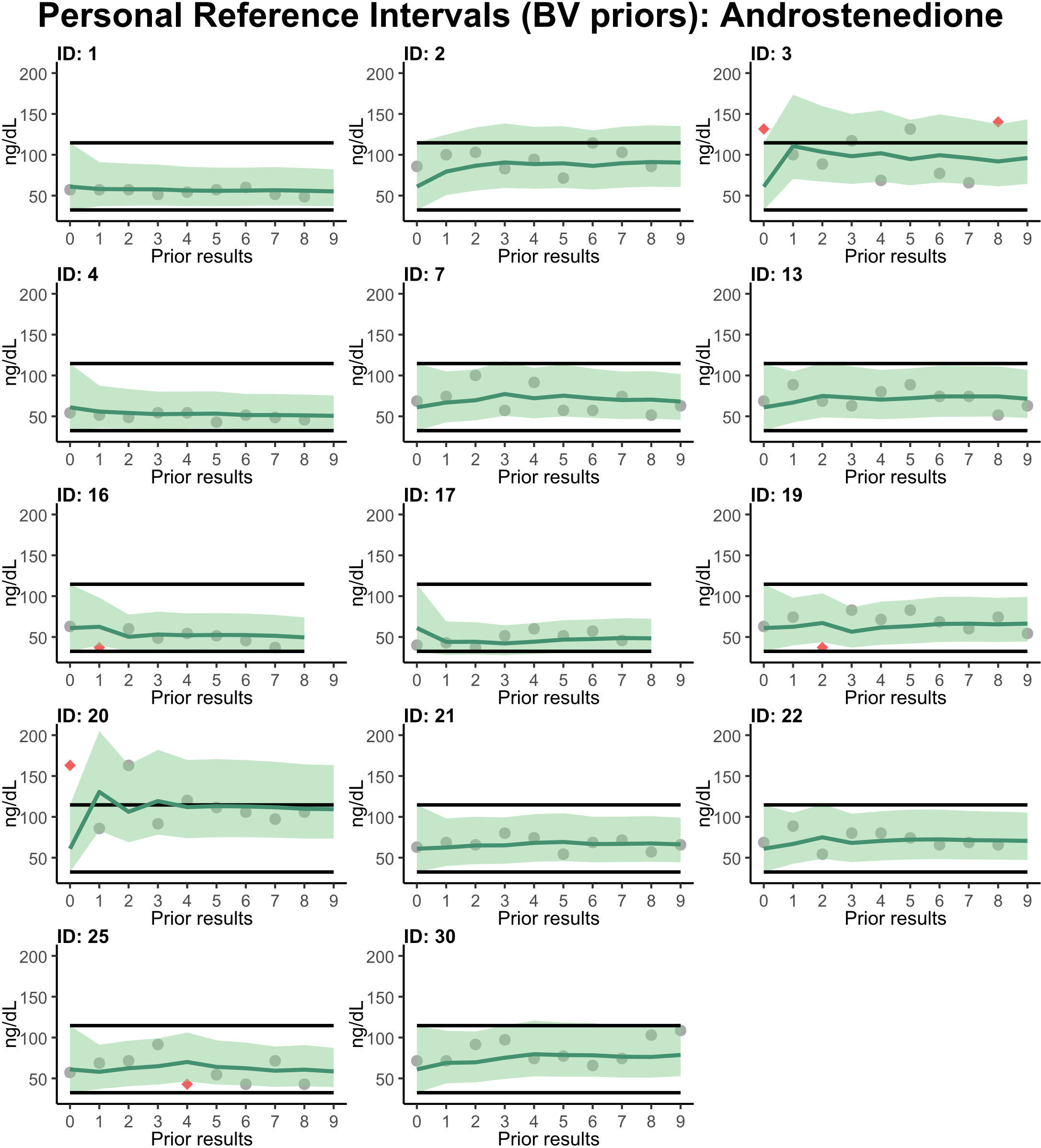

**Suppl. Figure 23:** Personalized reference intervals for androstenedione (RI_per_), with a two-sided 95% prediction interval (shaded green area), using PEB parameters based on biological variation estimates, across measurements from male participants (dots) in the biological variation study. Horizontal black lines represent the 95% reference interval determined by the refineR algorithm, while the red diamond denotes a flagged measurement exceeding the RI_per_.
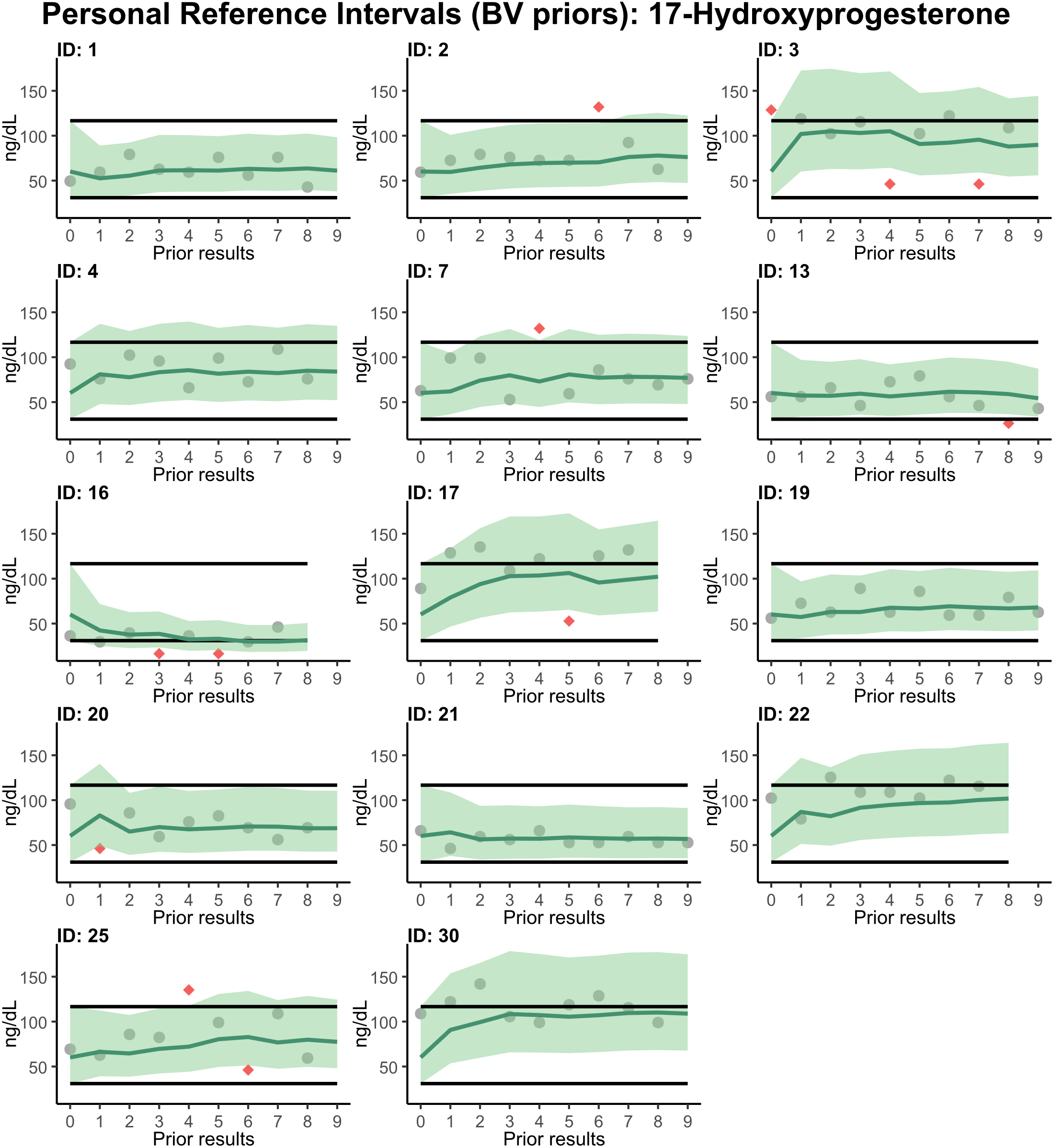

**Suppl. Figure 24:** Personalized reference intervals for 17-Hydroxyprogesterone (RI_per_), with a two-sided 95% prediction interval (shaded green area), using PEB parameters based on biological variation estimates, across measurements from male participants (dots) in the biological variation study. Horizontal black lines represent the 95% reference interval determined by the refineR algorithm, while the red diamond denotes a flagged measurement exceeding the RI_per_.
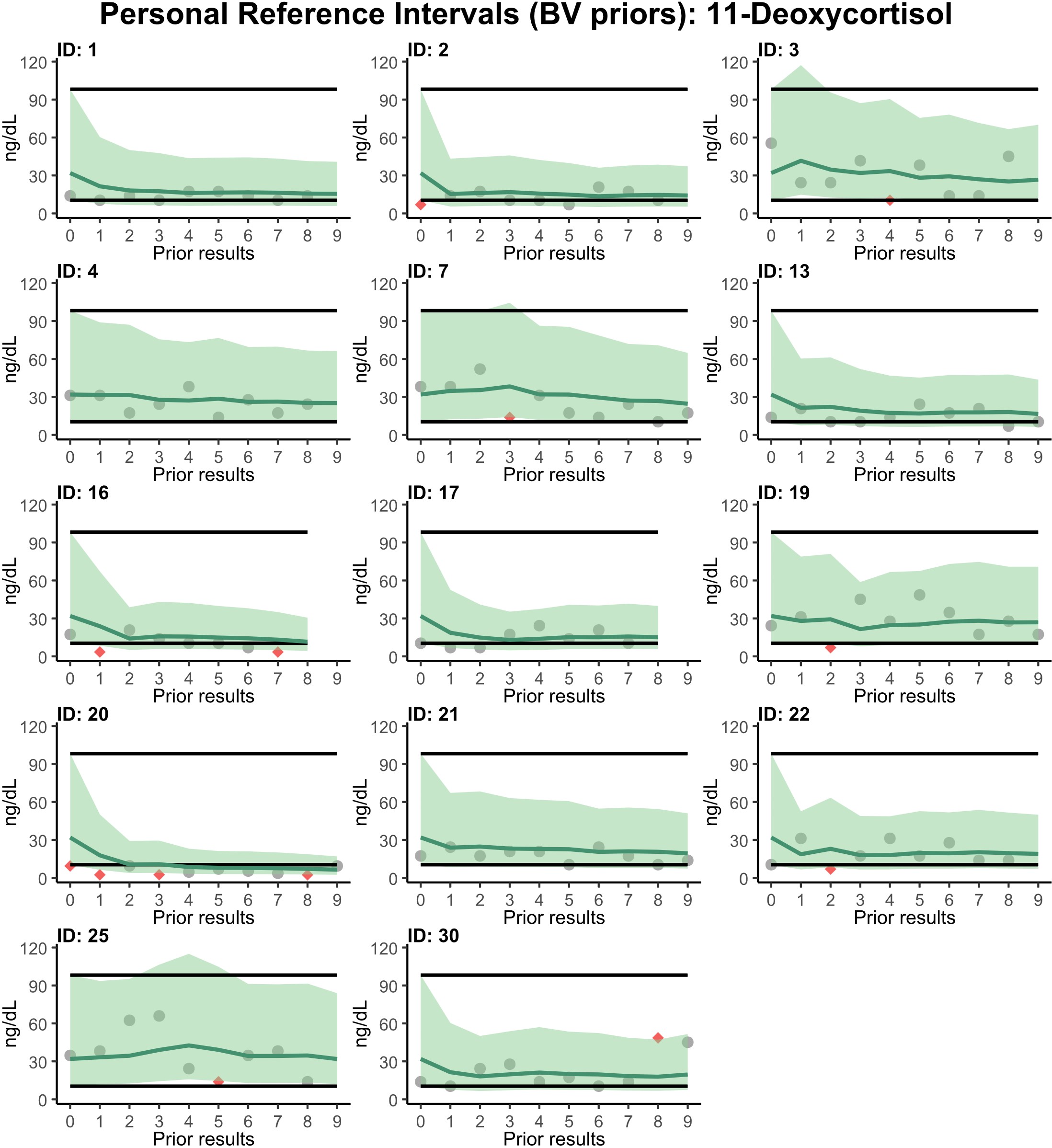
 **Suppl. Figure 25:** Personalized reference intervals for 11-Deoxycortisol (RI_per_), with a two-sided 95% prediction interval (shaded green area), using PEB parameters based on biological variation estimates, across measurements from male participants (dots) in the biological variation study. Horizontal black lines represent the 95% reference interval determined by the refineR algorithm, while the red diamond denotes a flagged measurement exceeding the RI_per_.

**Personalized Reference Intervals (BV-parameters): Testosterone**

**Personalized Reference Intervals (BV-parameters): Androstenedione**

**Personalized Reference Intervals (BV-parameters): 17-Hydroxyprogesterone**

**Personalized Reference Intervals (BV-parameters): 11-Deoxycortisol**


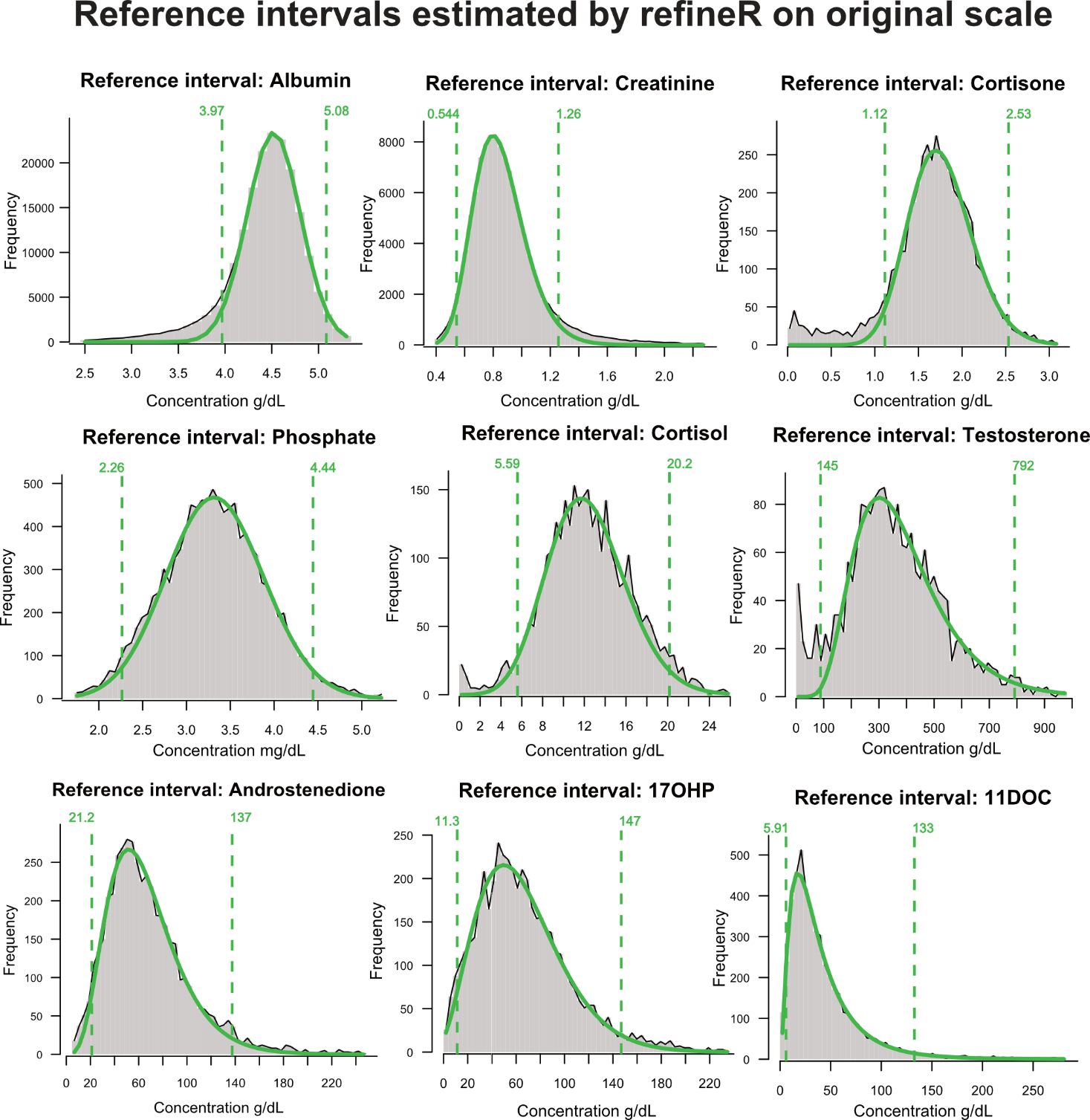


**Reference Intervals estimated by refineR on original scale**

**Suppl. Figure 26:** Reference intervals (vertical green lines) estimated by the refinerR algorithm fitted to the laboratory information system data (grey distribution) for the included biomarkers. 17OHP: 17-Hydroxyprogesterone, 11DOC: 11-Deoxycortisol.

**Suppl.**
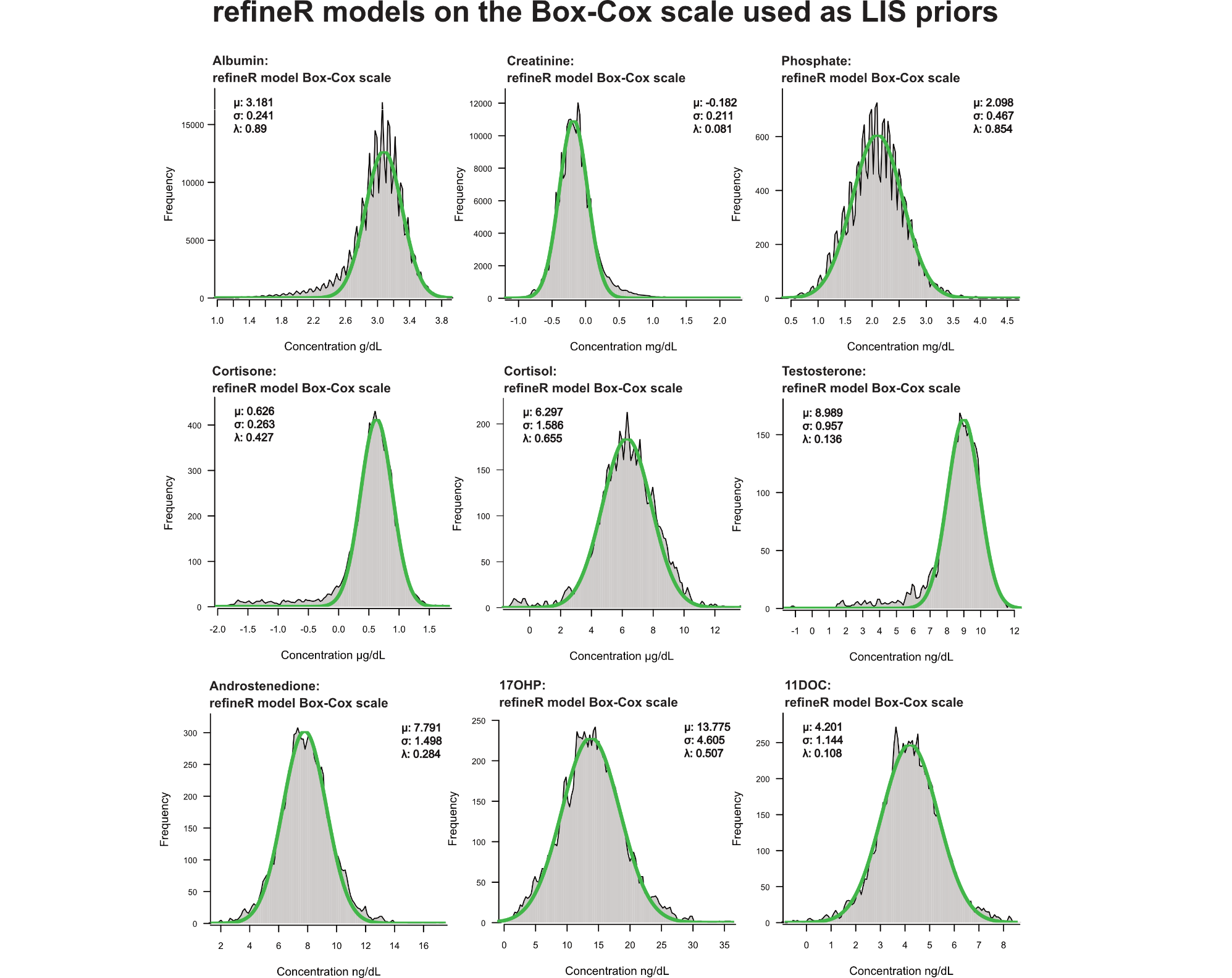
**Figure 27:** refineR models (green lines) on the Box-Cox scale, on which the estimated reference intervals are based. The refineR parameters μ and σ equal the population mean, and standard deviation on the Box-Cox scale used as PEB parameters. The λ specifies the Box-Cox transformation used to make the data approximate normality. 17OHP: 17-Hydroxyprogesterone, 11DOC: 11-Deoxycortisol.

**refineR models on the Box-Cox scale used to derive LIS parameters**


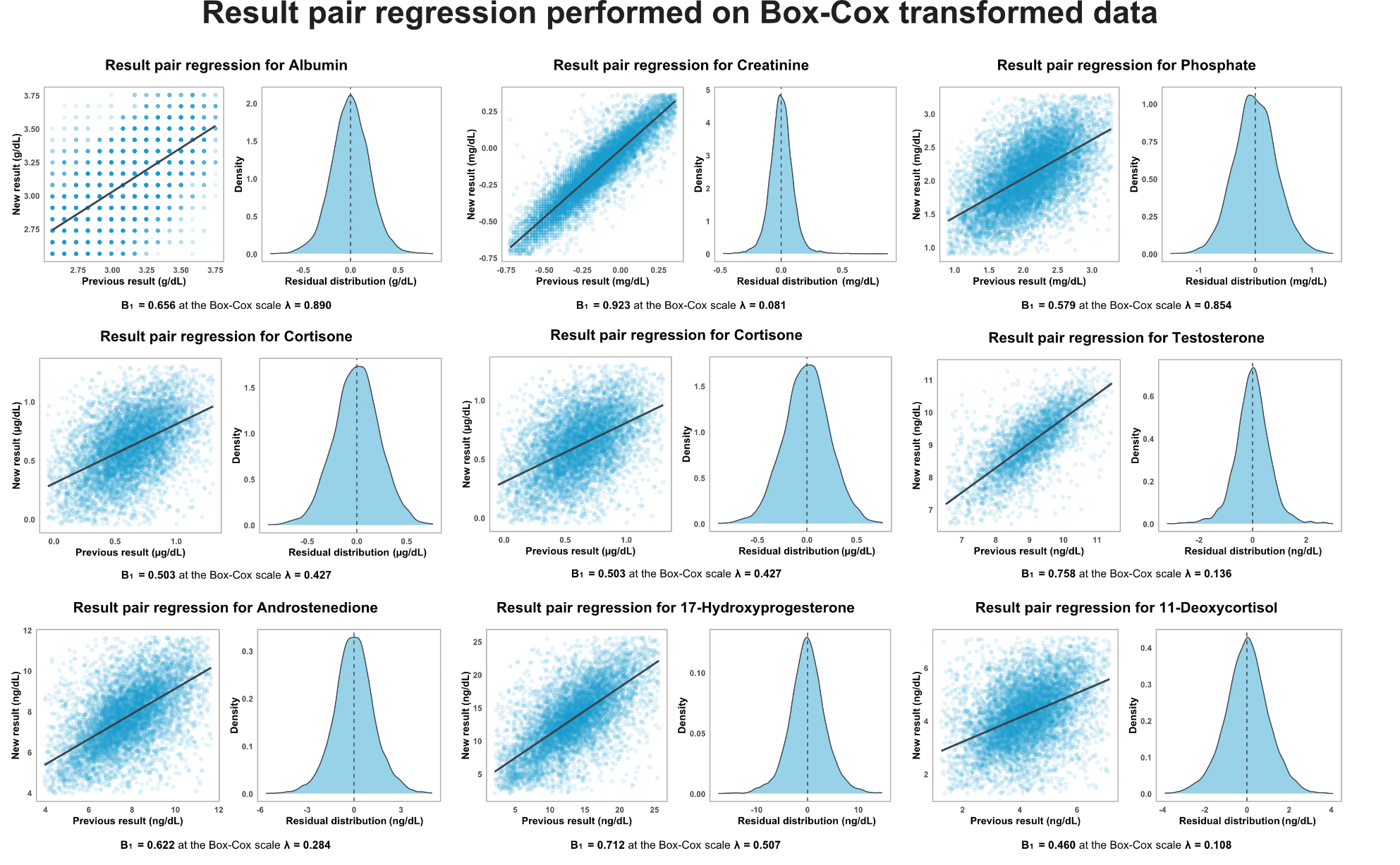


**Suppl. Figure 28:** The intraclass correlation (ICC), estimated as the slope (B_1_) of the robust regression applied to result pairs from the laboratory information system (LIS) data, was determined on the Box-Cox transformed scale for the included biomarkers. The kernel density distribution of the residuals was examined to confirm the assumption of normality.
