# Supplementary material for: A Parametric Empirical Bayes Approach to Personalized Reference Intervals and Reference Change Values
Source: Clin Chem. 2025 Aug 22;71(11):1147–57. doi: 10.1093/clinchem/hvaf092 (PMC12582661; doi:10.1093/clinchem/hvaf092)
Supplement: hvaf092_Supplementary_Data [file hvaf092_supplementary_data.zip › Supplemental formulas.docx]

**Supplemental formulas**

**Box-Cox transformation (1) and back-transformation (2)**

$$\mathbf{y=}\left\{ \begin{aligned} \frac{x^{\lambda}-1}{\lambda}, if \lambda\neq0 \\ \log\left( x \right), if \lambda=1 \end{aligned} \right. (1)$$

$$\mathbf{y=}\left\{ \begin{aligned} {(x \cdot\lambda+1)}^{\frac{1}{\lambda}}, if \lambda\neq0 \\ \exp\left( x \right), if \lambda=1 \end{aligned} \right. (2)$$

| $\mathrm{CV}_{\mathrm{total}}= \sqrt{\mathrm{CV}_{I}^{2}+\mathrm{CV}_{A}^{2}}$ | **Step 1** |
| --- | --- |
| $\sigma_{\mathrm{total}}=\sqrt{\ln\left( 1-{\mathrm{CV}_{\mathrm{total}}}^{2} \right)} , \sigma_{G}=\sqrt{\ln\left( 1-{\mathrm{CV}_{G}}^{2} \right)}$ | **Step 2** |
| $\sigma_{\mathrm{pop}}= \sqrt{\sigma_{\mathrm{total}}^{2}+ \sigma_{G}^{2}}$ | **Step 3** |
| $B_{1} = \frac{\sigma_{G}^{2}}{\sigma_{G}^{2}+ \sigma_{\mathrm{total}}^{2}}$ | **Step 4** |
| $B_{n}= \frac{B_{1}\cdot n}{B_{1}\cdot n+(1-B_{1})}$ | **Step 5** |
| $\mu_{\mathrm{pop}}=\ln\left( \mu_{\mathrm{pop}} \right), \bar{X}_{n} =ln \left( \bar{X}_{n} \right)$ | **Step 6** |
| $Ŷ=\mu_{\mathrm{pop}}+(\bar{X}-\mu_{\mathrm{pop}}){\cdot B}_{n}$ | **Step 7** |
| $\exp\left( Ŷ- Z \cdot\sqrt{1-B_{1}\cdot B_{n}}\cdot\sigma_{\mathrm{pop}} \right){\leq X}_{n+1}\leq\exp\left( Ŷ+ Z \cdot\sqrt{1-B_{1}\cdot B_{n}}\cdot\sigma_{\mathrm{pop}} \right)$ | **Step 8** |

**Parametric empirical Bayes thresholds from coefficients of variation**

**1.** Calculate the total variation for results of individuals (CV_Total_) by combining the within-subject variation (CV_I_) and local analytical variation (CV_A_) **2.** Calculate the standard deviations on the log scale from CV_Total_ and between-subject variation (CV_G_) **3.** Calculate the σ_pop_ by combining these standard deviations on the log-scale **4.** Calculate the intraclass correlation (B_1_)​ **5.** Calculate the shrinkage factor (B_𝑛_) based on B_1_ and the number of prior individual results (𝑛) **6.** Log-transform the population mean (μ_pop_) and individual sample mean (X̄_𝑛_, calculated form 𝑛 previous observations) **7.** Calculate the predicted homeostatic set point (Ŷ) **8.** Calculate the PEB thresholds at a specified significance level (determined by the Z-score) before back-transforming to the original scale. Check if the subsequent individual result X_𝑛+1_ exceeds the thresholds. For each new observation, recalculate the threshold for X_𝑛+1_ by updating 𝑛 and X̄_𝑛_ in steps **5-8**.**Proof 1: The shrinking factor (B**_𝑛_**) can be expressed in terms of the intraclass correlation (B_1_) and the number of previous samples (𝑛).**

*Assumption:* In the current and following proofs we assume σ_G_^2^ > 0 and σ_I_^2^ > 0 and the intraclass correlation satisfies B_1_ ∈ (0,1). We want to show that the shrinkage factor $B_{n}= \frac{\sigma_{G}^{2}}{\sigma_{G}^{2}+\frac{\sigma_{I}^{2}}{n}}$ can be expressed in terms of the intraclass correlation $B_{1}$ and the number of previous samples 𝑛.

Starting with the definition $B_{1}= \frac{\sigma_{G}^{2}}{\sigma_{G}^{2}+\sigma_{I}^{2}}$ we multiply both sides with ${(\sigma}_{G}^{2}+\sigma_{I}^{2})$:

$B_{1}\cdot\left( \sigma_{G}^{2}+\sigma_{I}^{2} \right)= \frac{\sigma_{G}^{2}}{\sigma_{G}^{2}+\sigma_{I}^{2}} \cdot\left( \sigma_{G}^{2}+\sigma_{I}^{2} \right)=B_{1}\sigma_{G}^{2}+B_{1}\sigma_{I}^{2}=\sigma_{G}^{2}$ ,

moving $\sigma_{G}^{2}$ to the right we get:

$${B_{1}\sigma}_{I}^{2}=\sigma_{G}^{2}-B_{1}\sigma_{G}^{2} \Longrightarrow{B_{1}\sigma}_{I}^{2}=\sigma_{G}^{2}\left( 1- B_{1} \right)$$

Isolating $B_{1}$ we this expression:

$$\frac{B_{1}}{\left( 1- B_{1} \right)}=\frac{\sigma_{G}^{2}}{\sigma_{I}^{2}}$$

We now rewrite $B_{n}$ in terms of $\frac{\sigma_{G}^{2}}{\sigma_{I}^{2}}$by multiplying the by numerator and denominator of$B_{n}$ by $\frac{1}{\sigma_{I}^{2}}$:

$B_{n}= \frac{\sigma_{G}^{2}}{\sigma_{G}^{2}+\frac{\sigma_{I}^{2}}{n}} \cdot\frac{\frac{1}{\sigma_{I}^{2}}}{\frac{1}{\sigma_{I}^{2}}}=\frac{\frac{\sigma_{G}^{2}}{\sigma_{I}^{2}}}{\frac{\sigma_{G}^{2}}{\sigma_{I}^{2}} +\left( \frac{\sigma_{I}^{2}}{n} \right) \cdot\frac{1}{\sigma_{I}^{2}}}= \frac{\frac{\sigma_{G}^{2}}{\sigma_{I}^{2}}}{\frac{\sigma_{G}^{2}}{\sigma_{I}^{2}} + \frac{1}{n}}$

Substitute $\frac{\sigma_{G}^{2}}{\sigma_{I}^{2}}$ with the expression of $B_{1}$ derived above we get:

$B_{n}= \frac{\frac{B_{1}}{\left( 1- B_{1} \right)}}{\frac{B_{1}}{\left( 1- B_{1} \right)}+ \frac{1}{n}}$

Finally, we can simplify the fraction by multiplying numerator and denominator by $\left( 1- B_{1} \right)\cdot n$. This step clears the fractions inside the denominator, but is only valid for $n\geq1$:

$B_{n}= \frac{\frac{B_{1}}{\left( 1- B_{1} \right)}}{\frac{B_{1}}{\left( 1- B_{1} \right)}+ \frac{1}{n}} \cdot\frac{\left( 1- B_{1} \right)\cdot n}{\left( 1- B_{1} \right)\cdot n} = \frac{B_{1}\cdot n}{B_{1}\cdot n +\left( 1- B_{1} \right)}$

For the case of $n= 0$ we consider that $B_{n}$ is a function of $n$ and consider the finite limit $n\to0^{+}$.

$$\lim_{n\to0^{+}} B_{n}=B_{0}= \frac{B_{1}\cdot0}{B_{1}\cdot0 +\left( 1- B_{1} \right)}=0$$

From this limit we see that $B_{n}$ approaches 0 as $n\to0^{+}$. This relies on the above assumption of $B_{1}\in(0,1)$ which makes $({1-B}_{1})$ strictly positive. Including the limiting behavior, we can define $B_{0}=0$ and $B_{n}$ as:

$$B_{n}= \frac{B_{1}\cdot n}{B_{1}\cdot n +\left( 1- B_{1} \right)}, \mathrm{for} n\geq0$$

Showing that $B_{n}$ can be written purely in terms of $B_{1}$ and $n$ for all $n\geq0$

**Proof 2: PEB transition from population reference intervals (RI_pop_) to personal reference intervals (RI_per_) depending on the number of previous results:1)** From “Proof 1” we define B_0_ = 0. With this definition the threshold for first individual measurement (𝑛 = 0 previous samples) solves to the RI_pop_ when using the PEB formulas:
$B_{0}= \frac{B_{1}\cdot0}{B_{1}\cdot0 + (1-B_{1})}=0$, so

${Ŷ=\mu_{\mathrm{pop}}+\left( \bar{X}_{n}- \mu_{\mathrm{pop}} \right)\cdot B}_{0}= \mu_{\mathrm{pop}}+\left( 0 -\mu_{\mathrm{pop}} \right)\cdot0 = \mu_{\mathrm{pop}}$, the threshold then becomes:

$$\left| X_{0+1}-Ŷ \right|> Z \cdot\sqrt{1-B_{1}\cdot B_{0}}\cdot\sigma_{\mathrm{pop}}= \left| X_{1}-\mu_{\mathrm{pop}} \right|>Z \cdot\sqrt{1-B_{1}\cdot0}\cdot\sigma_{\mathrm{pop}}$$

$\left| X_{1}-\mu_{\mathrm{pop}} \right|>Z \cdot\sigma_{\mathrm{pop}}$, which is equal to the RI_pop_**2)** In contrast, when 𝑛 **→ ∞ t**he PEB threshold converges toward the RI_per_:

$B_{\boldsymbol{\infty}}= \frac{B_{1}\cdot\infty}{B_{1}\cdot\infty+ \left( 1-B_{1} \right)}\approx1$, so

$${Ŷ=\mu_{\mathrm{pop}}+\left( \bar{X}_{n} - \mu_{\mathrm{pop}} \right)\cdot B}_{\boldsymbol{\infty}}\approx\mu_{\mathrm{pop}}+\left( \bar{X}_{n} - \mu_{\mathrm{pop}} \right)\cdot1= \bar{X}_{n}, the threshold then becomes:$$

$$\left| \bar{X}_{n+1}- Ŷ \right|> Z \cdot\sqrt{1-B_{1}\cdot B_{\boldsymbol{\infty}}}\cdot\sigma_{\mathrm{pop}}= \left| \bar{X}_{n+1}-\bar{X}_{n} \right|>Z \cdot\sqrt{1-B_{1}}\cdot\sigma_{\mathrm{pop}}$$

$$We can simplify this expression further:$$

$B_{1}=\frac{\sigma_{G}^{2}}{\sigma_{G}^{2}+ \sigma_{I}^{2}} \mathrm{and} \sigma_{\mathrm{pop}}= \sqrt{\sigma_{G}^{2}+ \sigma_{I}^{2}} so we can express \sqrt{1-B_{1}}\cdot\sigma_{\mathrm{pop}} \mathrm{as}$:

$$\sqrt{\left( 1-\frac{\sigma_{G}^{2}}{\sigma_{G}^{2}+ \sigma_{I}^{2}} \right)\cdot\sigma_{G}^{2}+ \sigma_{I}^{2}}= \sqrt{\left( \frac{\sigma_{G}^{2}+\sigma_{I}^{2}}{\sigma_{G}^{2}+\sigma_{I}^{2}}-\frac{\sigma_{G}^{2}}{\sigma_{G}^{2}+ \sigma_{I}^{2}} \right)\cdot\left( \sigma_{G}^{2}+ \sigma_{I}^{2} \right)}=$$

$\sqrt{\left( \frac{\sigma_{I}^{2}}{\sigma_{G}^{2}+ \sigma_{I}^{2}} \right)\cdot\left( \sigma_{G}^{2}+ \sigma_{I}^{2} \right)}= \sqrt{\sigma_{I}^{2}}= \sigma_{I},$ which creates the simplified threshold:

$\left| \bar{X}_{n+1}-\bar{X}_{n} \right|>Z \cdot\sigma_{I}$, which is equal to the RI_per_

 **Proof 3: Parametric Empirical Bayes < Reference Change Values**Assuming a normal distribution, the reference change value (RCV) combines the standard deviation of the within-subject biological variation σ_I_ and analytical variation σ_A_ as: Z ⋅ √2 ⋅ √(σ_I_^2^ + σ_A_^2^). The level of σ_A_ does not impact the proof since we can view the total variance (σ_I_^2^ + σ_A_^2^) as an increased σ_I_. We consider the σ_A_ = 0 for simplicity, leaving Z ⋅ √2 ⋅ σ_I_.
We derive the PEB threshold for 𝑛 = 1 to be:

$$Z \cdot\sqrt{1-{B_{1}}^{2}} {\cdot\sigma}_{\mathrm{pop}}, where B_{1}= \frac{\sigma_{G}^{2}}{\sigma_{G}^{2}+ \sigma_{I}^{2}}\mathrm{and}{\sigma_{\mathrm{pop}}}^{2}= \sigma_{G}^{2}+ \sigma_{I}^{2}$$

To prove that the PEB threshold is narrower than the RCV we must show that PEB < RCV:
$\sqrt{1-{B_{1}}^{2}} {\cdot\sigma}_{\mathrm{pop}} < \sqrt{2}\sigma_{I}$ We start solving this inequality by squaring both sides, so we obtain PEB^2^ < RCV^2^:

$$\left( 1-{B_{1}}^{2} \right){\sigma_{\mathrm{pop}}}^{2} < 2\sigma_{I}^{2}$$

Then expanding 1 - B_1_^2^:

$$1-\left( \frac{\sigma_{G}^{2}}{\sigma_{G}^{2}+ \sigma_{I}^{2}} \right)^{2}=1-\frac{\sigma_{G}^{4}}{{{(\sigma}_{G}^{2}+ \sigma_{I}^{2})}^{2}}=\frac{{{(\sigma}_{G}^{2}+ \sigma_{I}^{2})}^{2}}{{{(\sigma}_{G}^{2}+ \sigma_{I}^{2})}^{2}}-\frac{\sigma_{G}^{4}}{{{(\sigma}_{G}^{2}+ \sigma_{I}^{2})}^{2}}= \frac{{{(\sigma}_{G}^{2}+ \sigma_{I}^{2})}^{2}- \sigma_{G}^{4}}{{{(\sigma}_{G}^{2}+ \sigma_{I}^{2})}^{2}}$$

And rearranging the numerator:

$${{(\sigma}_{G}^{2}+ \sigma_{I}^{2})}^{2}- \sigma_{G}^{4}=\sigma_{G}^{4}+{2\sigma}_{G}^{2}\sigma_{I}^{2}+\sigma_{I}^{4}- \sigma_{G}^{4}= \sigma_{I}^{2}\left( {2\sigma}_{G}^{2}+\sigma_{I}^{2} \right)$$

We now get 1 - B_1_^2^ expressed as:

$$\frac{\sigma_{I}^{2}\left( {2\sigma}_{G}^{2}+\sigma_{I}^{2} \right)}{{{(\sigma}_{G}^{2}+ \sigma_{I}^{2})}^{2}}$$

We substitute this value for 1-B_1_^2^ back into our formula for PEB and get:

$$\frac{\sigma_{I}^{2}({2\sigma}_{G}^{2}+\sigma_{I}^{2})}{{{(\sigma}_{G}^{2}+ \sigma_{I}^{2})}^{2}} \cdot{(\sigma}_{G}^{2}+ \sigma_{I}^{2})= \frac{\sigma_{I}^{2}({2\sigma}_{G}^{2}+\sigma_{I}^{2})}{\sigma_{G}^{2}+ \sigma_{I}^{2}}$$

We can now use the expanded expression to show that PEB^2^ is < RCV^2^:

$$\frac{\sigma_{I}^{2}\left( {2\sigma}_{G}^{2}+\sigma_{I}^{2} \right)}{\sigma_{G}^{2}+ \sigma_{I}^{2}}< 2\sigma_{I}^{2}$$

First, we simplify by dividing both sides by σ_I_^2^:

$$\frac{{2\sigma}_{G}^{2}+\sigma_{I}^{2}}{\sigma_{G}^{2}+ \sigma_{I}^{2}}< 2$$

Then multiplying both sides by σ_G_^2^ + σ_I_^2^:

$${2\sigma}_{G}^{2}+\sigma_{I}^{2}< 2{(\sigma}_{G}^{2}+ \sigma_{I}^{2})$$

We now get that PEB^2^ / RCV^2^ < 1

$$\frac{{2\sigma}_{G}^{2}+\sigma_{I}^{2}}{2{(\sigma}_{G}^{2}+ \sigma_{I}^{2})}<1$$

As both σ_G_^2^ and σ_I_^2^ will always be > 0 we now see that the PEB^2^ must be < RCV^2^, we see this clearly if we simplify the expression further:

$${2\sigma}_{G}^{2}+\sigma_{I}^{2}<2{(\sigma}_{G}^{2}+ \sigma_{I}^{2})$$

$${2\sigma}_{G}^{2}+\sigma_{I}^{2}<2\sigma_{G}^{2}+ {2\sigma}_{I}^{2}$$

$$\sigma_{I}^{2}<{2\sigma}_{I}^{2}$$

$$1<2$$

To determine how much smaller, we can consider the PEB to RCV ratio by square rooting the PEB^2^ / RCV^2^ expression above:

$$\sqrt{\frac{{2\sigma}_{G}^{2}+\sigma_{I}^{2}}{2{(\sigma}_{G}^{2}+ \sigma_{I}^{2})}}$$

Three limiting cases illustrate the range of this ratio.
In the case of σ_G_^2^ → 0 the PEB / RCV shows that PEB is ≈ 30% smaller than the RCV:

$$\sqrt{\frac{\sigma_{I}^{2}}{2\sigma_{I}^{2}}}= \frac{1}{\sqrt{2}}\approx0.707$$

In the opposite scenario σ_I_^2^ → 0 the PEB / RCV shows that the PEB converges toward the RCV:

$$\sqrt{\frac{2\sigma_{G}^{2}}{2\sigma_{G}^{2}}}= 1$$

In the case of σ_G_^2^ → ∞ (or σ_I_ → ∞, or both) PEB / RCV shows that the PEB converges toward the RCV:

$$\sqrt{\frac{2\cdot\infty^{2}+\sigma_{I}^{2}}{2(\infty^{2}+ \sigma_{I}^{2})}}\approx1$$

In conclusion, the PEB threshold is between 0% and approximately 30% smaller than the RCV threshold, depending on the values of σ_G_^2^ and ​σ_I_^2^:

$$\frac{1}{\sqrt{2}}<\frac{\mathrm{PEB}}{\mathrm{RCV}} <1$$
